# “The Creative Force of Mathematical Formulations”: Werner Heisenberg and the Past, Present, and Future of Quantum Theory

**DOI:** 10.3390/e28060676

**Published:** 2026-06-11

**Authors:** Arkady Plotnitsky

**Affiliations:** Literature, Theory, and Cultural Studies Program, Philosophy and Literature Program, College of Liberal Arts, Purdue University, West Lafayette, IN 47907, USA; plotnits@purdue.edu

**Keywords:** continuity, creative force, discontinuity, Platonism, reality without realism, symmetry

## Abstract

This article offers a new argument concerning the relationships between mathematics and physical reality in quantum theory. While, as dedicated to the centenary of Heisenberg’s invention of quantum mechanics, the article contains a historical discussion, it is not historical, but physical and philosophical. Heisenberg’s thinking leading him to QM and beyond is only part of the genealogy of this argument, which also differs from that of Heisenberg and builds on the previous work of the present author. Among the contributions in the article are a new conception of the ultimate reality responsible for quantum phenomena; a new understanding, based on this conception, of the role of mathematics in physics in quantum theory; a critique of Heisenberg’s and all Platonism in quantum theory and beyond; a new perspective on the role of symmetries in quantum field theory; and a new understanding of the relationships between continuity and discontinuity in quantum physics.

The Pythagoreans seem to have been the first to realize the creative force inherent in mathematical formulations.

—Werner Heisenberg [1] (p. 64)

## 1. Introduction

This article is derived from the paper given at the special session (organized by this author), celebrating the centenary of Werner Heisenberg’s invention of quantum mechanics (QM), at the 2025 conference on quantum foundations, “Quantum information and probability: From foundations to engineering” (the Linnaeus University, Växjö, Sweden, 10–14 June 2025). The article is a contribution to the proceedings of this conference to which this Special Issue of *Entropy* is devoted. While, however, the article, in view of this genealogy, contains significant historical content, it is not a historical article. It offers a new physical and philosophical argument concerning the relationships between mathematics and physical reality in quantum theory. Heisenberg’s ideas, leading him to his invention and beyond, are only part of the genealogy of this argument, which also, in some respects, importantly differs from Heisenberg’s argumentation and offers a critique of the latter by building on the previous work of the present author. Among the main contributions in this article are a new conception of the ultimate reality responsible for quantum phenomena (Section 2); a new understanding, based on this conception, of the role of mathematics in physics in view of quantum theory (Section 3); a critique of Heisenberg’s and by implication all Platonism in quantum theory and beyond (Section 4); a new perspective on the role of symmetries in quantum field theory (QFT) (Section 5); and a new understanding of the relationships between continuity is discontinuity in quantum physics (Section 6).

Heisenberg’s invention of QM, which, a century later, remains the standard theory of quantum phenomena in nonrelativistic regimes, was a revolutionary transformation of modern physics as a mathematical–experimental science, which physics became, with René Descartes, Galileo, and others, in the seventeenth century. This article offers a new physical and philosophical understanding of this transformation. This understanding is grounded in the new type of relationship between a physical theory and physical reality, arising from Heisenberg’s mathematical thinking, leading him to QM, which gave an even greater significance to the role of mathematics as governing the conjunction of the mathematical and experimental in all modern physics, from Descartes and Galileo on. This relationship was not necessarily assumed by Heisenberg himself, even at the time of his discovery and especially in his later thinking, in part shaped by QFT, thinking that eventually, by the 1940s, led him to a form of what I shall define as “materialist Platonism.” The present view of this relationship originates in Niels Bohr’s thinking, emerging in the wake of Heisenberg’s discovery and defining his interpretation in all its versions (there were several), reaching its ultimate version around 1937. This view is based on the concept of “reality without realism” (RWR) and is hereafter referred to as the RWR view, which leads to the corresponding RWR interpretations, considered by this author previously most extensively in [2]. Bohr’s interpretations, except possibly the one in the Como lecture of 1929 [3] (v. 1, pp. 51–93), are RWR interpretations. There can be more than one such interpretation, and the one advanced here is different from that of Bohr. The main precursor of the RWR view itself was, in addition to that of Bohr, Henri Lebesgue in his response to the logical complexities of Georg Cantor’s set theory. The RWR view, thus also defining the concept of RWR, places the ultimate reality responsible for quantum phenomena beyond representation (hereafter the weak RWR view) or even beyond conception and thus the reach of human thinking (hereafter the strong RWR view), in the latter case even to the point of this reality being neither material nor mental. This is a new conception introduced in this article, not considered by this author previously, or by Bohr or Lebesgue. Bohr, while adopting an RWR interpretation of QM, assumed this reality to be material. So did Heisenberg, even in his Platonist stage, thus making his Platonism materialist. While there is no evidence that Lebesgue entertained an idea of reality that is neither material nor mental, his view more readily allows for this possibility by suggesting that something that we cannot conceive of might nevertheless exist [4] (pp. 261–273). The RWR interpretation adopted here is only assumed to be *an interpretation*: it is an interpretative inference responding to the mathematical–experimental structure of quantum physics, as different from the mathematical–experimental structures of classical physics and relativity. It is not a metaphysical assertion about nature as existing apart from us, although, as discussed below, it is possible that the ultimate reality responsible for quantum phenomena is an RWR-type reality, which may also be neither material nor mental.

Mathematics was the primary creative force of Heisenberg’s thinking in his invention of QM and all his work. The phrase “the creative force of mathematics” comes from Heisenberg, according to whom: “The Pythagoreans seem to have been the first to realize the creative force inherent in mathematical formulations” [1] (p. 64). While this sentence occurs in his later *Physics and Philosophy*, where it is linked to his materialist Platonism, it equally defines his view and practice in his invention of QM. As discussed below, it is not certain whether Heisenberg at the time assumed a form of the RWR view, as Bohr expressly did, or thought along the lines of materialist Platonism even then.

QM and QFT, in their standard versions, are the only quantum theories to be considered in this article, and, unless qualified, the term quantum theory will refer to them. Quantum phenomena are assumed to be defined by the fact that, in considering them, the role of Planck’s constant, *h*, must be taken into account, while this role can be neglected in classical physics or relativity. This assumption may require qualifications, which can be put aside, because all quantum phenomena considered here are defined by the role of *h* in them [5] (pp. 37–38) [6] (pp. 52–56). Quantum phenomena could be interpreted separately from any theory accounting for them. On the other hand, an interpretation of any such theory, like QM or QFT, must involve an interpretation of quantum phenomena, which the RWR interpretations considered in this article, including the one adopted by it, do as well. Classical physics also commonly refers to classical physical theories, such as classical mechanics, classical statistical physics, or classical electromagnetic theory, and relativity to relativity theory. I shall retain this use, qualifying when referring to experimental classical or relativistic physics. Technically, a different interpretation of a theory forms a different theory, because an interpretation may involve concepts not shared by other interpretations. For simplicity, however, I shall refer to interpretations of QM or QFT. By contrast, by classical physics and relativity, I shall refer to theories themselves. The reason for doing so is that most interpretations of these theories, including here, are realist or ontological, insofar as the reality responsible for the phenomena considered by these theories is assumed to be represented by them. Although the uses of “realist” and “ontological” sometimes diverge, they are close and will be used, as adjectives, interchangeably here. I adopt “realism” as a noun, as a general term, and “ontology” as a noun, referring to specific representations or conceptions of the reality considered. By contrast, the proliferation of different, including incompatible, interpretations of QM or QFT has been massive, and debates concerning them have continued with an undiminished intensity for a century now.

The dominant role of mathematics in Heisenberg’s thinking and work has been extensively discussed, although it will be given a greater significance here as a creative force and a new philosophical understanding. His physics have been extensively discussed as well. Considering the transformative philosophical aspects of Heisenberg’s thinking is less common. Doing so is additionally complicated by the fact that Heisenberg primarily discussed his philosophical views in his later writings from a standpoint of materialist Platonism, different from the one adopted by him at the time of his discovery of QM. His philosophical standpoint then was at least more open to the RWR view, incompatible with materialist Platonism. The latter assumes that the ultimate nature of reality considered is represented purely mathematically, in the absence of physical concepts. It was then and has remained an uncommon position, although it has some affinities with, and may even be seen as a form of (ontic) structural realism, developed later [7]. Among the differences between them are that Heisenberg does not subscribe to the idea of “relations without relata,” often adopted in structural realism, and that he deals with higher-level mathematical structures, such as infinite-dimensional group representations, than structural realism does. RWR interpretations and materialist Platonist ones do, however, share the assumption that the ultimate reality responsible for quantum phenomena cannot be represented by physical concepts, only applicable to quantum phenomena themselves. The difference is that in RWR interpretations, this reality, as beyond representation or even conception, cannot be represented mathematically either. As will be seen, however, Heisenberg’s materialist Platonist interpretation of QM or QFT was accompanied by the Aristotelian idea of “*potentia*,” which is not mathematical and, as such, poses difficulties for materialist Platonism.

It is more common, in considering the founding figures of QM, to associate philosophical thinking, especially that along nonrealist lines, concerning QM with Bohr. Heisenberg did so himself, beginning with invoking, in honoring Bohr’s contribution, “the Copenhagen spirit of quantum theory [*der Kopenhagener Geist der Quantentheorie*]” [8] (p. iv) and introducing the term the Copenhagen interpretation in [1]. The term, however, designated a particular interpretation, which was his own, even if adopting some features of Bohr’s various interpretations. There were, thus, more than one “Copenhagen interpretation” even in Bohr’s or Heisenberg’s cases. The term has by now encompassed a large set of different and sometimes incompatible interpretations, and it will not be used here. While warranted, the juxtaposition of the mathematical and philosophical orientation of Heisenberg’s and Bohr’s thinking, respectively, requires qualifications. Apart from the fact that Bohr reflected on the new mathematics of QM and a new way in which mathematics was used [9], the *functioning* of his key concepts, such as complementarity, which are not mathematical, depends on how mathematics works in QM. Conversely, Heisenberg’s thinking, either leading him to his discovery of QM or in his later works, contained philosophical features transcending the mathematics of QM. Also, while influenced by Bohr, Heisenberg’s philosophical views were different from those of Bohr, especially in Heisenberg’s later writings. Importantly, too, Heisenberg’s thinking, leading to his discoveries of QM and then the uncertainty relations, in turn impacted Bohr’s philosophical thinking. It is more transparent and better known that Heisenberg’s discovery of the uncertainty relations was crucial for Bohr’s invention of complementarity, introduced in the wake of this discovery [10] (pp. 191–193, 220–232). Heisenberg’s philosophical ideas, however, that led to his discovery of QM influenced Bohr’s philosophical thinking as well.

As noted, I will not be primarily concerned here with biographical and historical aspects of Heisenberg’s discovery of QM or his subsequent work. While these aspects cannot be entirely bypassed, a sustained analysis of them would belong to an investigation different from the one, primarily physical and philosophical in character, undertaken here. It is true, or is in any event the present view, that the history of physics shapes physics, more so than most physicists or even philosophers of physics realize. The role or the character of this history may, however, be different in different cases, and considered differently or with a different emphasis. Following G. W. F. Hegel’s view of the defining role of concepts and their history in philosophy, the invention and history of concepts take the center stage in this article, primarily physical and mathematical concepts, but also philosophical concepts, conjoined with physical and mathematical ones. The concept of concept, often taken for granted, is discussed in Section 2. For the moment, the invention of a new concept belongs to the thinking of a particular figure (or several figures), such as that of Heisenberg’s new quantum variables. The process of this invention is, however, only partially available, because access to this thinking is limited even when the author is alive and could provide as much information as possible concerning this thinking, or if the author had left an extensive record of this thinking. Thus, some key ideas in Heisenberg’s discovery of QM are often assumed to have come to him while, suffering from hay fever, he retreated to the island of Helgoland in June 1925. Narratives associated with this event, beginning with Heisenberg’s own recollections, made the word “Helgoland” a symbol of Heisenberg’s discovery. These narratives, including Heisenberg’s own (decades after the event), have been challenged, revealing “Helgoland” to be more of a legend, often used to support a common myth of a great discovery made single-handedly via a divine-like revelation by a great scientist. Although there may be some truth to the Helgoland legend or to this myth, it is hard to be certain how much truth there is. In any event, while these considerations are more important in biographical or historical accounts, they are secondary to the present philosophical argument. As concerns Heisenberg’s thinking, this argument is based on his works and allows one to avoid historical difficulties concerning the exact time or place of specific events, such as what happened where at the time of Heisenberg’s discovery of QM.

The primary history considered here is the history of concepts, and every concept, no matter how innovative, has a history in other concepts, rather than only in specific personal or social circumstances of its invention. These circumstances may have a significant bearing on the nature of concepts. I am not advocating any Platonism or idealism of concepts as existent in themselves and by themselves somewhere and discovered by human thought entirely independently of the historical and cultural circumstances at the time. In this article, however, as physical and philosophical in character, these circumstances will only be addressed when necessary for understanding the physical and philosophical nature and functioning of the concepts considered, either those of Heisenberg and others, or those of this article itself, such as that of reality without realism (RWR). This article is a *critique* of all Platonism, idealist or materialist. This critique is not a dismissal. I use the term critique in the sense of Immanuel Kant’s critical philosophy, as an analysis of conceptual structures, which leads toward a deeper understanding of them and retaining some of their aspects, while removing others, in order to create new conceptual structures. Heisenberg pursued such a critique (and used the term in this sense) in developing new concepts of quantum theory by means of a critique of those of classical theory [8] (pp. 13–54). While, however, it remains fertile in this respect or in developing new concepts from its concepts, Platonism may also stand in the way of new concepts developed otherwise. It has been and remains dominant in mathematical or scientific thinking, whether so named, as mathematical Platonism, or not, as in most forms of realism, materialist or idealist, which share with Platonism the view, defining it, that *it is possible to conceive of and even represent that which is*. This is not possible in RWR interpretations in the case of the ultimate reality responsible for quantum phenomena, but only in the case of these phenomena themselves, which, while described classically, can be predicted by quantum theory. The RWR thinking follows Platonism, from Plato to Albert Einstein and Heisenberg (in his Platonist phase) and beyond, in giving mathematics a primary role in our *interactions* with reality in quantum physics. This thinking, however, irrevocably breaks with all Platonism by precluding mathematics from representing or enabling one to form a conception of this reality, because it precludes any form of thought from doing so.

## 2. “Experimental Because of Its Mathematical Project”: Modern Physics from Descartes to Heisenberg and the Structure of Quantum Theory

Taking as its starting point Martin Heidegger’s insight concerning the mathematical character of modern physics, this section considers how QM, while retaining and even enhancing this character, changes it by transforming, following Heisenberg’s and Bohr’s thinking, our understanding of the ultimate reality responsible for quantum phenomena. It is this transformation that leads the present author to understand this reality as reality without realism (RWR). “Modern science,” Heidegger argues in his analysis of René Descartes’s and Galileo’s physics, “is experimental because of its mathematical project [*Entwurf*]” [11] (p. 93). It has indeed been so defined from Descartes and Galileo to relativity and quantum physics, and beyond, and still is. Heisenberg’s approach to QM and then QFT gave mathematics an even greater role in the mathematical–experimental project of modern physics, thus defined, in two alternative ways, corresponding to two stages of Heisenberg’s thinking about quantum theory. The first is defined by the RWR view and the corresponding interpretations of QM or QFT, beginning with that of Bohr. These interpretations place the ultimate reality responsible for quantum phenomena beyond any representation or, in their strong form, beyond conception, physical or mathematical, to the point (beyond Bohr’s view) of this reality being neither material nor mental. The second is defined by the materialist Platonism of Heisenberg’s later thinking, in which this reality is represented by the mathematics of quantum theory in the absence of physical concepts, at least as conventionally understood, from the rise of classical physics on. These concepts are equally excluded in representing or even conceiving of this reality in RWR interpretations, along with mathematical concepts. The new type of mathematics of quantum theory, introduced by Heisenberg, nevertheless, played a key role in and in part led to the RWR view of quantum physics, just as this mathematics later led Heisenberg to materialist Platonism. There are also more conventional realist interpretations, based on (mathematized) physical concepts, in accord with what is sometimes known as scientific realism, the dominant philosophical desideratum among physicists and philosophers. These interpretations will, however, be only briefly discussed here by way of contrast with RWR and materialist-Platonist interpretations, as contrasting alternatives to this conventional realism.

Heisenberg’s mathematics of QM remains his greatest invention, arguably even more significant than that of the (Heisenberg) uncertainty relations, with which his contribution and name have been more commonly associated. Although, as with every concept or theory, Heisenberg’s formalism had its history, including in Heisenberg’s own earlier work on, as it became known after QM, “the old quantum theory,” this formalism was entirely unprecedented in physics. Matrix algebra was known in mathematics by then. Heisenberg was famously unaware of its existence and reinvented it; technically, he invented a mathematically isomorphic algebra because he did not think of his variables as matrices but as double-indexed ensembles of quantities. Heisenberg’s use of this mathematics had a key precursor in the idea, entirely unprecedented as well, of Bohr’s 1913 atomic theory [12], that of “quantum jumps,” as discontinuous transitions between stationary states, in which electrons had the same energy. While in Bohr’s theory, stationary states were represented classically by orbital motions of electrons around nuclei, the transitions between them were not given a mechanical representation, which (along with accompanying postulates) brought Bohr’s theory into conflict with both classical mechanics and classical electrodynamics [12] (p. 7). Heisenberg’s theory not only retained this workable, but unwelcome, feature, but it also extended it by abandoning the orbital motion of electrons altogether, in part because it was unobservable, as *motion* (an electron’s position in a stationary state could be observed). Thus, Heisenberg’s theory no longer represented, in terms of physical motion of objects in space and time, the ultimate reality responsible for quantum phenomena, observed in the instruments used, and the data contained there. This absence of a physical representation still allowed for a purely mathematical representation, eventually adopted by Heisenberg in his materialist Platonism. These data were assumed, initially implicitly, to be represented by classical physics, which could not predict them. This incapacity became apparent beginning with Max Planck’s analysis of black body radiation, which led Planck to a quantum alternative, manifested in his famous law, in 1900. Overcoming limitations of the old quantum theory, QM was able to predict these data for all (nonrelativistic) quantum phenomena. It could only do so probabilistically, even in the case of the simplest individual quantum systems, which was and remains strictly in accord with quantum experiments. No other predictions are possible because identically prepared quantum experiments generally lead to different outcomes, or as Bohr said, “different recordings,” observed in measuring instruments [3] (v. 2, p. 73).

Heisenberg’s approach thus suggested a new, twofold structure of physical reality in quantum physics and a new architecture of quantum theory. This structure was expressly formulated by Bohr and developed by him over the next decade as part of his interpretation, ultimately (he changed his view several times) as a strong RWR interpretation. In this interpretation, while probabilistically predicting the data contained in quantum phenomena, QM no longer represented either quantum phenomena or these data, represented by classical physics, or the ultimate reality responsible for quantum phenomena, which was not represented at all and was eventually, around 1937, assumed by Bohr to be beyond conception. The possibility of this type of interpretation made Heisenberg’s QM revolutionary philosophically, rather than only physically and mathematically. This aspect of Heisenberg’s theory was, again, anticipated in Bohr’s 1913 theory in the case of quantum jumps [12]. Heisenberg’s QM, however, extended this to all quantum events, making them “quantum jumps,” and introduced an entirely new type of mathematics in dealing with this twofold architecture of physical reality. Heisenberg termed his new variables, defining this mathematics, “new kinematics,” a misapplied designation because these variables did not represent the motion of quantum objects. The recourse to these variables reflected the difficulties of applying the concept of motion to quantum objects and avoided these difficulties by abandoning this concept. The equations of QM were no longer equations of motion. By replacing physical kinematics with his *mathematical* kinematics, Heisenberg made mathematics the *dynamics of thinking* about quantum theory and changed the nature of theoretical physics in dealing with quantum phenomena.

In the process, mathematics acquired an even greater role in QM than in modern physics previously, fundamentally mathematical, including in governing its experimental nature, as it has been from its rise on, as, to return to Heidegger’s characterization, “experimental because of its mathematical project.” This is the case not only or even primarily because of the role of quantitative measurement in modern physics, vs. Aristotle’s physics as a physics of qualitative observations, although this difference was essential to this mathematical–experimental project. Measurement was a mathematical concept and functioned as such in modern physics, on the model extending from ancient Greek geometry, which was a science (*episteme*) of physical space and measurements in space, geo-*metry*, which made it a form of physics, and a model, even the model for future sciences on this account, as well as that of its logical rigor. Aristotle’s *physics* was, however, an essentially qualitative theory of motions of entities in either domain, material or mental, although it did contain some formal laws and quantitative elements. The primary reason for Heidegger’s claim was that modern physics used mathematics to relate and, until quantum theory, to represent the reality considered, by finding a mathematically idealized representation of this reality and, by means of this idealization, to predict the data found in experimentally observed phenomena. Geometry still played the primary role in this representational project, both practically and philosophically. Sir Issac Newton was compelled to present his mechanics in terms of Euclidean geometry rather than calculus (by means of which he discovered it) in his 1687 *Principia* [13], in part, as he explained, to assure a geometrical demonstration of his findings. While calculus was not considered, and was not established as, mathematically legitimate at the time, using calculus was also about thinking more algebraically, as especially manifested in Leibniz’s more formal version of it. Accordingly, algebra was far from absent in modern physics either. Nevertheless, QM gave algebra a new and more dominant role in physics as well, compelling Einstein to see “the Heisenberg method” as purely “algebraic” [14] (p. 378). As explained later, this assessment was not entirely accurate, because geometry, in a more abstract form and with a new role in physics, was present in this “method,” defined primarily by the creative role of mathematics in theoretical physics, equally Einstein’s own method.

After Heisenberg, this method has become pervasive and was even pronounced by Paul Dirac to be “the most powerful method” of advancing at least quantum theory [15] (p. 1). Among the founding figures, Dirac’s thinking was the closest to Heisenberg in making this creative force drive quantum theory, with an even greater emphasis on formal mathematical thinking. In this respect, Dirac exercised a strong influence, arguably stronger than that of Heisenberg, on the subsequent approaches to QM and QFT. Sometimes this method was taken to the point of virtually making theoretical physics mathematics, only indirectly referring to the experiment, and the case of string and M-brane theories was criticized on this account. I am taking a more open view concerning these theories. Excessively mathematical as they may be, they are more in accord with than depart from modern theoretical physics as defined by the creative force of mathematical thinking.

I am not claiming that mathematics is always the primary creative force in theoretical physics. It was not mathematics, but the invention of new physical concepts that led Bohr to the concept of quantum jumps and then complementarity, his most famous concept, or Einstein to special relativity or even to general relativity (GR). Mathematics took over Einstein’s work on GR and his subsequent unsuccessful attempts to combine it with electromagnetism. Bohr, for whom the main creative force was that of the invention of new physical concepts, was an exception in quantum theory or even twentieth-century theoretical physics. Heisenberg was more a rule than an exception. His accomplishment in the creation of QM, enabled by his mathematical creativity, was exceptional, rivaling the achievements of his greatest precursors. What distinguished his use of mathematics was that this mathematics was divorced from representing, via mathematized physical concepts, the ultimate reality responsible for quantum phenomena. It was restricted to probabilistic predictions concerning the data observed in these phenomena. This was true in Bohr’s theory of quantum jumps, but Bohr’s mathematics was essentially the same as that of classical physics. A purely mathematical representation, apart from physical concepts, on lines of materialist Platonism, was in principle allowed (hence my above qualification), as possibly assumed by Heisenberg even then. Heisenberg used some mathematics common in physics, such as Fourier analysis. The key mathematics of QM was, however, linear algebra and functional analysis, by then developed in mathematics but not used in physics.

Making mathematics independent of physics, from roughly 1800 onward, defines what is now called modern mathematics. Modern physics refers to mathematical–experimental physics, from roughly 1600 onward. The rise of modern physics tied mathematics to physics. These ties were broken by modern mathematics, although algebra had developed as independent from physics, from around 1600 on, becoming ever more abstract in modern mathematics, eventually, contemporaneously with the rise of quantum theory, becoming the study of formal algebraic structures, known as “modern algebra.” Group theory, which became crucial for QM and QFT, was developed in this way earlier. Heisenberg’s approach to QM returned modern mathematics, in effect defined by algebraic structures, such as operator algebras (Lie algebras), to physics in a new way by using this mathematics for probabilistic predictions of the outcomes of quantum experiments rather than for describing the ultimate reality responsible for what is observed in these experiments. While Heisenberg did not define his variables in these terms, his thinking and even more so that of Dirac may be seen as abstract-algebraic in the same sense. Thus, Heisenberg’s mathematical thinking was not helped by a mathematical refinement of physical concepts, as in classical physics or (with qualifications) relativity. On the other hand, this thinking was free from the constraints imposed by such a representation. Heisenberg’s materialist Platonism was also based on a purely mathematical representation of the ultimate reality considered, apart from physical concepts, using that which defines most forms of realism in physics. There are, as noted, exceptions, such as ontic structural realism [7].

The creation of new concepts is the primary aim of the creative mathematical or physical drives considered here. Such drives could be directed otherwise, for example, as in quantum logic, to a deeper understanding of the logical structure of QM, which might also involve new concepts. There is no single concept of concept, and the term is often taken for granted in scientific or even philosophical literature. The concept of concepts that I shall adopt in part follows that of Gilles Deleuze and Félix Guattari’s concept of a *philosophical* concept but also departs from it [16] and [17] (pp. 52–76). They juxtapose their concept of concept, referring strictly to philosophical concepts, with mathematical and scientific concepts. They even deny that mathematics or science has concepts in their sense. “The concept,” they say, “belongs to philosophy and only to philosophy” [16] (pp. 11–12, 33–34). They see mathematics and science as defined by logical propositions and formal structures, which are composed of elements without being concepts in their sense. Logic and calculations are, of course, essential to mathematics or science. I would argue, however, that Heisenberg’s new quantum variables or Bohr’s complementarity, or even a function, *f* (*x*), are concepts in the sense akin to that of Deleuze and Guattari and have an analogously primary role in *creative* thinking in mathematics and science. The difference is that mathematical and scientific concepts have a technical exactitude that philosophical concepts, while rigorous in their own way, do not. The importance of concepts is not limited to creative aspects of mathematics or science. Working with and teaching established concepts is indispensable in mathematics or science. Logical and calculational thinking can lead to the creation of new concepts as well.

In the present understanding, a concept is not merely a generalization from particulars (which commonly defines concepts in linguistics or cognitive sciences) or a general or abstract idea, although a concept may contain such ideas. A concept is a multicomponent structure, defined by the organization, *composition*, of its components, and some of these components may be concepts in turn. What meaningfully defines a concept is its specific composition, determined by each component and the nature of its relations within this composition. In fact, there are no simple concepts. A single-component concept is a product of a provisional cut-off of its multicomponent organization. A concept is always a composition. Heisenberg’s variables were composed of specific elements and in specific relations to each other within double-indexed arrays (in effect, matrices). Not all components of a concept need to be new in a new concept, but their composition must be new to make a concept new. As stressed from the outset, every concept, no matter how innovative, has a history defined by earlier concepts and depends on them.

For Heisenberg, however, the creative force of mathematics was a way of dealing with the experimental evidence, rather than being, first, a mathematical “play,” as it was, at least to a greater degree, for Dirac, who even referred to his work as “playing with equations” [18]. Heisenberg saw his emphasis on the experiment as indebted to Bohr. This may appear surprising because Bohr’s thinking was more concerned with the excess, seen by him as irreducible, of mathematics in considering the ultimate reality responsible for quantum phenomena. Heisenberg’s materialist Platonism, eliminating physical concepts in representing this reality, would not have been acceptable to Bohr. Nevertheless, the experiment was the defining concern for both Bohr and Heisenberg at all stages of their thinking, including when Heisenberg adopted materialist Platonism. In reflecting on Bohr’s influence in this regard, Heisenberg emphasized Bohr’s philosophical thinking: “Bohr was primarily a philosopher, not a physicist, but he understood that natural philosophy in our day and age carries weight only if its every detail can be subjected to the inexorable test of experiment” [19] (p. 95). I would question that Bohr was *primarily* a philosopher. I would argue that, while he was both, he was primarily a physicist, and his philosophy, even when it moves beyond physics, comes from his thinking about physics, whatever the philosophical history of his physical concepts [5] (pp. 111–121). Heisenberg’s statement disregards Bohr’s creative drive toward the invention of new concepts, such as complementarity and later phenomenon, or earlier quantum jump, one of the most revolutionary physical concepts ever. While these concepts have philosophical dimensions, they are physical and were necessary for physical reasons, two of them, in particular. The first was the experimental evidence on which these concepts were based, such as the stability of atoms in the case of quantum jumps, the uncertainty relations, or what is classically observed on measuring instruments, in the case of complementarity and phenomena. The second was Bohr’s RWR interpretation, grounded in these concepts, ultimately, by around 1937, in its strong RWR form, placing the ultimate reality responsible for quantum phenomena beyond conception, vs. merely placing it beyond representation or knowledge, the weak RWR view, assumed by him previously. Most RWR interpretations considered in this article, including the one it adopts, are strong, and unless qualified, “RWR interpretations,” “RWR thinking,” or “the RWR view” refer to their strong forms.

Heisenberg’s statement does, however, convey an important aspect of the relationships among mathematics, physics, and philosophy, in Bohr, which was adopted by Heisenberg, while giving a greater weight to mathematics in these relationships. Heisenberg’s discovery of QM was defined by the attitude described in the sentence immediately preceding the one on Bohr cited above: “Thus I understood: knowledge of Nature was *primarily* obtained in this way [by intense preoccupation with the actual phenomena], and only as the next step can one succeed in fixing one’s knowledge in mathematical form and subjecting it to complete rational analysis” [19] (p. 95). Heisenberg’s thinking always begins with the experimental evidence, notwithstanding his Platonist inclinations, found even at the time of his discovery of QM. This entails no inconsistency with the primacy of the creative force of mathematics in this thinking, and the role of abstract mathematics there, but it gives both the fundamental groundings in experiment, as confirmed by Heisenberg’s accounts of his thinking leading to the invention of QM. Thus, shortly before finishing his paper containing his discovery of QM, he wrote to Ralph Kronig:

What I really like in this scheme is that one can really reduce all interactions between atoms and the external world to transition probabilities.

(Letter to R. Kronig, 5 June 1925; cited in [20], v. 2, p. 242).

Grounded in the physical, experimentally established interactions between “atoms and the external world,” this “reduction” required a mathematical “scheme” that could predict these probabilities. Finding this scheme, as QM, was Heisenberg’s greatest invention. The transition probabilities predicted by this “scheme” were those between the events of the interactions between atoms and the external world, by using observational instruments, without physically representing what happens between these events.

What were Heisenberg’s reasons to “really like” this reduction, given that it was a diminishment of what classical physics or relativity were able to do in representing the behavior of their objects? First, this representation became difficult because the interactions between the atoms and the external world, defined, in quantum experiments, by the measuring instruments used, were constitutive of quantum phenomena and could not be controlled and hence taken into account, or neglected, as in classical physics or relativity. Accordingly, the representational structure of these theories could not be adopted in mathematically dealing with quantum phenomena. Secondly, while it had major successes, the old quantum theory, which offered a partial representation of the behavior of atomic objects, had run into major difficulties by the 1920s. One way to deal with these difficulties was to have a theory dispensing with physically considering these interactions or, in the first place, the physical description of quantum objects and reduced to predicting transition probabilities between observed quantum phenomena, defined by these interactions. It was the theory that had to be so reduced rather than these interactions. As Bohr said in his assessment of “the new quantum mechanics” in the wake of Heisenberg’s and Max Born and Pascual Jordan’s paper, which gave Heisenberg’s “scheme” its proper matrix form [21], but before Schrödinger’s wave mechanics:

[A] In contrast to ordinary mechanics, the new quantum mechanics does not deal with a space–time description of the motion of atomic particles. [B] It operates with manifolds of quantities which replace the harmonic oscillating components of the motion and symbolize the possibilities of transitions between stationary states …. These quantities satisfy certain relations which take the place of the mechanical equations of motion and the quantization rules [of the old quantum theory].

[3] (v. 1, p. 48)

Part (B), which refers to Heisenberg’s main mathematical innovation, will be discussed in the next section. Here, I shall focus on part (A). While representing Bohr’s view, as the RWR view, (A) may not have represented that of Heisenberg even at the time. The “reduction” of Heisenberg’s “scheme” to transition, as described in his letter to Kronig, still allowed that one could represent the ultimate reality responsible for quantum phenomena purely mathematically and apart from physical concepts, on lines of mathematical Platonism, rather than strictly entailing the RWR view, assumed by Bohr. I shall, nevertheless, following [22], refer to (A) as “the Heisenberg postulate,” because it was Heisenberg’s extension of Bohr’s handling of quantum jumps that led Bohr to (A). Matrix mechanics did not deal with electrons in stationary states, in which they maintained constant energy, but only with discontinuous transitions, “quantum jumps,” between them. Schrödinger’s time-dependent formalism and then the transformation theory of Dirac and Jordan (which combined both formalisms) were able to handle electrons’ behavior in stationary states, in which they would change their position. Heisenberg’s abandonment of the idea of the *unobservable* orbital motion of electrons in stationary states made these states just energy states, which were *observable*.

(A) only reflected part of Heisenberg’s thinking, as explained in Heisenberg’s letter to Kronig, but an epistemologically most radical part, at least allowing for the RWR view. Quantum phenomena, themselves, were now viewed as irreducibly discrete relative to each other, without assuming any continuous physical connections between them. This fact makes quantum phenomena, observed in the instruments used, irreducibly different from the quantum objects considered, regardless of interpretation. Nobody has ever seen an electron or photon, or any quantum object as such. It is only possible to observe traces of their interactions with suitable instruments, left in the observable parts of these instruments, such as spots on photographic plates, in accord with Heisenberg’s view of quantum physics, expressed in his letter to Kronig, as that of the “*interactions* between atoms and the external world.” This view was adopted by Bohr as a defining feature of his interpretation in all its versions.

From this vantage, Heisenberg’s thinking, leading him to his discovery of QM, suggested that QM may be understood in quantum-informational terms [2] (pp. 112–113, 310–318). The reason for this view is as follows. The quantum-mechanical situation, as Heisenberg conceived of it, was defined by:

(A) Certain already obtained information, derived from spectral lines (due to the emission of radiation by the electron), observed in measuring instruments.
(B) Certain possible future information, to be obtainable from spectral lines to be observed in measuring instruments and, hopefully, predictable in probabilistic or statistical terms by the mathematical formalism of a quantum theory.

Heisenberg aimed at developing this kind of formalism, without assuming that it needed to represent a spatiotemporal process connecting these two sets of information or how each comes about. This information was, in each case, determined by what type of experiment one decides to perform, rather than by measuring the preexisting properties of quantum objects, even individual such properties, rather than only certain jointed properties, as precluded by the uncertainty relations.

Bohr added another postulate, designated here as “the Bohr postulate”: the classical description of these effects and the observable parts of measuring instruments. Importantly, the latter were also assumed to have quantum parts enabling their interactions with quantum objects. The Bohr postulate thus defined is different from Bohr’s postulates of his 1913 theory, which are, in effect, underlain by the Heisenberg postulate applied to quantum jumps. Heisenberg adopted the Bohr postulate as well, thus assuming this twofold view of physical reality in quantum theory, even in his later thinking, governed by mathematical Platonism. RWR interpretations based on the Heisenberg postulate alone, without the Bohr postulate, are possible. Conversely, the Bohr postulate can be assumed by realist interpretations, as it was by Heisenberg’s materialist-Platonist interpretation. The RWR interpretation adopted in this article assumed both postulates but contains additional features. I would like, however, to give a more sustained outline of RWR interpretations before explaining these features in order to better ground them.

First, as in realist interpretations, the concept of reality without realism is based on more general concepts of reality and existence, assumed to be primitive concepts and not given analytical definitions. By “reality” I refer to that which is assumed to exist, without making any claims concerning the *character* of this existence, claims that define realism. Realist theories or interpretations aim to offer at least a conception but more commonly a representation of this reality, usually in terms of the objects considered by a theory. They also aim to predict the future course of this reality by using this representation, either ideally exactly, deterministically, as in classical mechanics of individual or simple systems or relativity (which is a deterministic theory), or probabilistically or statistically, as in classical statistical physics or chaos theory. The difference between probability and statistics will be put aside, although it may affect an interpretation, including an RWR one, of QM or QFT [5] (pp. 173–186). The absence of claims concerning the character of the reality considered or a stratum of this reality allows one to place this reality or this stratum beyond representation or even conception, as in the case of the ultimate reality responsible for quantum phenomena in RWR interpretations. I understand existence as a capacity to have effects on the world. The world may be defined, following Ludwig Wittgenstein, as “all that is the case, …the totality of facts, not of things,” and, thus, is real in our experience [23] (p. 24). A rigorous inference that something exists can only be made on the basis of its effects. RWR interpretations allow for or even entail representations of such effects as manifested in quantum phenomena; they only preclude a representation or conception of how these effects are ultimately possible.

RWR interpretations do not assume a unified character of this reality, only manifesting itself differently in each experiment. This assumption is incompatible at least with strong RWR interpretations, which preclude any conception of this reality and, hence, that of its unity. While each time unthinkable, an RWR reality is each time unique, manifesting its uniqueness in each encounter with this reality as an effect, each time unique in turn. More generally, as stated from the outset, the assumption of an RWR reality is only associated here, as *an interpretive assumption*, with quantum phenomena as ultimately responsible for this assumption in RWR interpretations. Indeed, it is impossible to assume, even for practical purposes, that this reality can be extracted, as independent, from the envelop of quantum phenomena. It is not a metaphysical assumption about nature. Nature is only assumed to exist independently of us, which amounts to the assumption that it existed before we existed and will continue to exist when we no longer exist. This assumption has been challenged, even to the point of denying that any material reality exists. Plato and Bishop Berkeley are the most famous cases, respectively ancient and modern, of this denial. Both, however, assumed the existence of spiritual reality independent of our existence. It is true that, as their argumentation usefully implies, any conception of how anything exists, or even that it exists, even as beyond thought, still belongs to thought. It does not follow, however, that something that our thought cannot conceive of does not exist.

This point was made in 1905 by Henri Lebesgue, one of the founders of modern integration and measure theory, in responding to the paradoxes of Georg Cantor’s set theory, shaking the foundations of mathematics then. Lebesgue argued that the fact that we cannot mathematically conceive of objects, “sets,” that are neither finite nor infinite, does not mean that such objects do not exist [4] (pp. 261–273). Similarly, that we cannot conceive of entities that are neither continuous nor discontinuous does not mean that such entities do not exist. Indeed, Lebesgue’s argument was made in response to the problem of the continuum and the debates concerning it, especially Cantor’s continuum hypothesis. The hypothesis states that there is no infinite set, the power of which is greater than that of a countable set and less than that of the continuum, defined as the number of points in a continuous line, such as the interval [0, 1]. The question of continuum was further problematized by Kurt Gödel’s incompleteness theorems of 1931 and Paul Cohen’s proof of the undecidability of the continuum hypothesis (the impossibility of mathematically proving it to be either true or false) in the 1960s. In view of these findings, we do not know and even cannot conceive how a continuous line is mathematically constituted by its points, and hence the ultimate constitution of continuity in mathematics. Lebesgue did not specify in what domain, material or mental, such entities might exist, but arguably, referred to either domain, if not to the possibility of a reality that is neither material nor mental, hence neither spatial nor temporal either. Assuming spatiality or temporality as properties of nature, rather than thought, has been challenged beginning with Kant, but under the assumption, including in most RWR interpretations, of the material nature of the reality considered, rather than of the possibility that this reality may be neither material nor mental. The concept of RWR, however, allows for this mere possibility in the case of the ultimate reality responsible for quantum phenomena, at least as an interpretive assumption.

For reasons discussed in detail in Section 4, the present interpretation adds another postulate, the Dirac postulate, according to which the concept of a quantum object, either in general or as concerns any specific type of it, such as an electron or a photon, is only applicable at the time of observation, and not to objects existing independently in nature. The latter is a more common, even nearly uniform, view, including that of Bohr or Heisenberg, although Heisenberg’s materialist Platonism in effect abandons it, as referring to material objects. Dirac adopts this view as well. The Dirac postulate is designated as such because, while not assumed by Dirac himself, the possibility of this assumption emerged with Dirac’s discovery of his equation for the relativistic electron, which revealed itself to be an equation for both the electron and the positron. It reflected and, as it happened, led to the discovery that, in high-energy quantum regimes, governed by QFT, the identity of a particle-*type* (rather than only particles themselves) could no longer be assumed in successive observations, as it could in classical physics, relativity, or nonrelativistic quantum physics. Thus, while the initial observation can register an electron, the next one can register a positron, a photon, or an electron–positron pair, with the probabilities defined by the same mathematical formalism. There are, however, also reasons to adopt the Dirac postulate in low-energy quantum regimes [22,24].

At stake in RWR interpretations is not merely dealing with the Kantian distinction between things-in-themselves (noumena), as the ultimate reality considered, and phenomena defined by our thinking, some representing exterior reality, material or mental [25]. Kant’s things-in-themselves can also be either. Instead, one deals with strata of this exterior reality, the first that of the ultimate reality, which, as the RWR-unthinkable, is moreover beyond conception, rather than, as in Kant, only knowledge; and the second is that of its effects, still exterior to phenomena and possibly material, but knowable and even representable by phenomena. At least this second stratum of reality can be treated as such for all practical purposes, as it could still be assumed to be akin to Kant’s things-in-themselves, workably phenomenally represented, while underlain by the RWR-unthinkable responsible for them, which cannot be so represented.

Importantly, the RWR-unthinkable does not refer to something that could at some future point be reached by thought; it refers to something that could *never* be reached by thought. This impossibility cannot be claimed with certainty because one cannot exclude that it will become possible to conceive of or even know the ultimate reality responsible for quantum phenomena. This impossibility can, however, be a *definitive interpretive* assumption, while leaving open whether this assumption is, or will remain, factually correct or not. In other words, if an RWR interpretation is adopted, there are two possibilities:

(A) This interpretation characterizes the situation in quantum physics at a given point in time, allowing that quantum phenomena or the theory predicting them, or whatever may replace it, will, at some future point, no longer require RWR interpretations, replacing them with realist interpretations, allowing for a conception or representation. Indeed, it is claimed by some that such is the case even now.
(B) This recourse to an RWR interpretation reflects the *possibility* that the ultimate reality responsible for quantum phenomena will never become available to thought, even if quantum theory acquires a different form or is replaced by an alternative theory, perhaps no longer quantum.

Thus, RWR thinking is not about replacing the RWR-unthinkable with the thinkable, as a new creation of thought that would replace this unthinkable. Doing so, or replacing what is unknown with new knowledge, is an important aspect of theoretical thinking, including when the RWR view is adopted. RWR thinking is, however, about creating new forms of thought and knowledge while assuming, at least for the practical purposes of an interpretation, a reality that is beyond the reach of thought and that will never be reached by thought. (A) would make RWR interpretations obsolete, as replaced by a realist theory or interpretation. This replacement, however, would not abolish the possibility of using RWR thinking in other situations, where it would be necessitated by different phenomena. It is possible, however, that there are phenomena, material or mental (either concept can apply to these phenomena, even if neither applies to the reality responsible for them) that would require (B), or even that there are in fact things in nature or thought itself, for example, in mathematics, that our thought can never reach. I am inclined to think that both cases are possible, even though neither can be certain. But neither can be (A), keeping in mind that in the present view, one only deals with RWR interpretations, alongside other possible interpretations, some of which are realist. There does not appear to be experimental or theoretical reasons to prefer either (A) or (B) in quantum physics. (A) and (B), however, reflect different expectations of how far our thought could reach in understanding the ultimate nature of reality, physical or mental, or possibly that which is neither.

Two key concepts defining classical physics and relativity, measurement and classical causality, become no longer applicable in quantum physics in RWR interpretations. In contrast to the classical concept of measurement, extending from the rise of geometry, geo-*metry*, in Ancient Greece to classical physics, in RWR interpretations, what is commonly referred to as a quantum measurement *does not measure*, or in the first place, *is not an observation of*, any property that the ultimate reality responsible for quantum phenomena would possess before or even during the act of observation, in which the interaction between this reality and the observational instrument used is transmuted to the classical reality of observation. Accordingly, the concept of observation requires redefinition as well. An act of observation in quantum physics establishes, *creates*, a quantum phenomenon by an interaction between the instrument used and the quantum object. What happens is *unavoidably* defined by what kinds of experiments we perform, by how we affect reality with our unique acts of creation. I qualify by “unavoidably” because the phenomena observed in classical physics or relativity may be affected by experimental technology, and we do stage different experiments there. Nevertheless, in principle, one can observe these phenomena without affecting what is observed because the interference of experimental technology is negligible or calculable. This also allows one to treat these phenomena as representing the corresponding objects and thus follow and represent what happens during and after this observation by disregarding this observation itself. This is never possible in quantum physics, regardless of interpretation, because the difference between quantum phenomena and quantum objects is irreducible. As an act of creation, an experiment defines a new reality and a possible future course of reality from this event on, making any preceding observation no longer meaningful in assessing this course. This fact as such does not exclude realist interpretations of QM, such as the many worlds one, or realist theories of quantum phenomena, such as Bohmian mechanics. In RWR interpretations, however, the ultimate reality responsible for quantum phenomena is beyond representation or even conception.

As such, these interpretations also preclude one from assuming “classical causality,” defining classical physics or relativity, and certain (unavoidably realist) interpretations of QM or QFT. By classical causality, often designated simply as “causality,” I refer to the claim that the state, X, of a physical system is determined, in accordance with a law, at all future moments of time once its state, A, is determined at a given moment of time, and A is determined by the same law by any of the system’s previous states, which can be reconstituted from A. This assumption implies a concept and at least a partial representation of the reality defining this law, making classical causality an ontological conception, which is incompatible with RWR interpretations. “Determinism” is sometimes used to designate classical causality. I use “determinism” as an epistemological category referring to the possibility of predicting the outcomes of classically causal processes ideally exactly. In classical mechanics, when dealing with individual or small systems, or in relativity, both concepts are coextensive. On the other hand, classical statistical mechanics or chaos theory are classically causal but not deterministic because the mechanical complexity of the systems considered limits us to probabilistic or statistical predictions.

The main reason for my choice of “classical causality,” rather than “causality,” is the possibility of alternative concepts of causality applicable in QM in RWR interpretations, where classical causality does not apply. Such a concept, “quantum causality,” was considered from the RWR perspective in [2] (pp. 207–218) [4] (pp. 203–207). For the present purposes, it is sufficient to give the main definition of the concept, defining its main difference from classical causality: an actual quantum event, A, defined by an act of observation, as an act of creation, accompanied by a measurement, allows one to predict which future events may happen with one probability or another, but, in contrast to classical causality, without assuming that any of these events will necessarily happen, *even in the absence of an outside interference or our capacity to predict them*. An outside interference can change what can happen even if the system obeys classical causality, which is, however, restored after this interference. Related concepts of causality were proposed in quantum information theory [26,27,28], apart, however, from RWR interpretations. The concept of quantum causality reflects the fact that RWR and some related interpretations (e.g., [28]) change the nature of probability in quantum vs. classical physics. In classical physics, the recourse to probability is merely practical due to our insufficient knowledge concerning the complex systems considered, while their elementary constituents behave classically causally and could be predicted ideally exactly. In quantum physics, in RWR interpretations, this recourse arises because there is no knowledge or even conception of how quantum phenomena come about [3] (v. 1, p. 34). Hence, no form of classical causality can apply, even when the phenomena concerned are associated with the simplest individual objects, such as elementary particles, thus precluding deterministic predictions even in these cases. According to Bohr:

[I]t is most important to realize that the recourse to probability laws under such circumstances is essentially different in aim from the familiar application of statistical considerations as practical means of accounting for the properties of mechanical systems of great structural complexity. In fact, in quantum physics we are presented not with intricacies of this kind, but with the inability of the classical frame of concepts to comprise the peculiar feature of indivisibility, or “individuality,” characterizing the elementary processes.

[3] (v. 2, p. 34)

The “indivisibility” refers to the indivisibility of phenomena in Bohr’s sense, defined, in Bohr’s or other RWR interpretations, by the impossibility of considering quantum objects independently of their interactions with these instruments. “Individuality” refers to the assumption that each quantum phenomenon is, in general, unrepeatable. It is the outcome of a unique act of creation, defined by the decision and possibly free will of the agent performing the experiment that gives rise to this phenomenon, thus defining reality at this moment in time, as opposed to locating an already established reality as an observation does in classical physics or relativity. Bohr eventually came to see quantum phenomena as revealing “a novel feature of *atomicity* in the laws of nature,” “disclosed” by “Planck’s discovery of the quantum of action [*h*], supplementing in such unexpected manner the old [Democritean] doctrine of the limited divisibility of matter” [29] (p. 94; emphasis added). Atomicity and, thus, discreteness or discontinuity initially emerged in quantum theory on this Democritean model, implicitly with M. Planck’s discovery of the quantum nature of radiation in 1900 and expressly with Einstein’s introduction of the concept of a photon, a *particle* of light, in 1906. QM, however, led Bohr to the concepts of phenomenon and atomicity, as defined strictly by what is observed in measuring instruments [3] (v. 2, p. 63). Bohr’s “atomicity” is essentially equivalent to that of phenomenon but highlights some of the features of the latter concept, such as individuality and discreteness.

It is this discreteness of observed quantum phenomena, rather than that of the ultimate reality responsible for them, that embodies the essential randomness of quantum physics. This kind of randomness is not found in classical physics, because all systems considered there, even those that are not deterministic, behave classically causally, and the behavior of the individual constitutive elements comprising these systems could, in principle, be predicted deterministically, usually by means of classical mechanics. There are situations, such as those of the Einstein–Podolsky–Rosen (EPR) type of experiments, where predictions concerning certain variables are ideally possible with probability equal to one. In view of the specific character of these predictions, however, they still do not entail classical causality [2] (pp. 207–218). It is crucial, however, that while quantum phenomena contain this randomness, they are not strictly random, but exhibit statistical correlations in certain circumstances, such as those of the Einstein–Podolsky–Rosen (EPR) type experiments. That this randomness of individual quantum events coexists with the statistical order of quantum correlations is one of the greatest mysteries of quantum physics, manifestly consistent, however, with RWR interpretations.

These interpretations give a central significance to the category of event. The concept of event assumed—(A) defining a new physical situation each time, (B) an effect of an RWR-type of reality observed in experimental technology, and (C) the creation of a quantum phenomenon—makes RWR interpretations, different from other event-based interpretations of QM or QFT, such as Carlo Rovelli’s relational interpretation [30] or that of Rudolph Haag [31], which are realist. In RWR interpretations, events are defined by experimental technology and our decisions concerning which experiment to perform. Each such decision creates a new reality and a new course of reality probabilistically predictable by QM or QFT, making it a form of decision science [32].

Bohr’s concept of complementarity makes this aspect of quantum physics especially pronounced and was in part designed to account for it. Defined most generally, complementarity entails

(A) A mutual exclusivity of phenomena (or possibly other entities, such as concepts).
(B) The possibility of considering, by decision, each one of them separately at any given point.
(C) The necessity of considering all of them at different moments of time for a comprehensive account of the totality of phenomena that one must consider in QP.

As are the uncertainty relations, complementarity is not a feature of quantum theory, but of quantum phenomena. QM and QFT are, however, fully in accord with complementarity, just as they are with the uncertainty relations, the primary case exemplifying complementarity. I might add that wave-particle complementarity, with which the concept of complementarity is often associated, had not played a significant, if any, role in Bohr’s argumentation [2] (pp. 205–206). Complementarity refers to the existence of incompatible situations of what is observed as individual quantum phenomena. The *possible* information concerning a quantum object, defined by the effects of the interactions between it and the instrument used, can only be obtained in mutually exclusive experiments, thus requiring them [3] (v. 2, p. 40). On the other hand, once made, either information, say, that of the position, is the complete *actual* information about the object, as complete as possible, at this moment in time. One could never obtain the complementary information—that concerning the momentum variable—at the same time because to do so one would need simultaneously to perform a complementarity experiment, which is impossible. In classical physics or relativity, one can obtain the ideally exact value of both variables in a single experiment. Hence, there are no uncertainty relations there.

By (B), above, however, one can always decide to perform either one or the other experiment and has a freedom or free will to do so, at least a degree of free will, which qualification does not diminish its significance. Complementarity is not only about the mutual exclusivity of the entities considered, but also about performing quantum experiments by human agents. One can set up a device to perform an experiment or treat a natural event, such as a radioactive emission, as an experiment. Any such setup is only possible by a human agent or by a device, such as a computer, which, as things stand now, must be programmed by a human agent to do so. That one has freedom, at least, a sufficient freedom, to decide which experiment to perform is, as Bohr says, in accordance with the very idea of experiment in science [33] (p. 699). In quantum physics, however, implementing our decision allows one to make only certain types of predictions, say, concerning future position measurements, and exclude certain other types of predictions, say, concerning future momentum measurements, in complementary situations. Complementarity, thus, exemplifies quantum causality because this future course could only be assessed probabilistically, which requires a mathematical theory. Hence, while complementarity as such is not a mathematical concept, its use in quantum physics requires mathematics, such as that of QM, which was in place, as were Heisenberg’s uncertainty relations, at the time Bohr introduced complementarity in 1927.

## 3. “The Most Powerful Method of Advance”: Heisenberg’s Mathematical Thinking and the Invention of Quantum Mechanics

This section discusses Heisenberg’s invention of the mathematics of QM and how this mathematics works in accord with the RWR view of the theory, whether Heisenberg himself adopted this or a similar view at the time or not. The next section considers how this mathematics works in materialist Platonism, adopted by Heisenberg in his late thinking, at least by the late 1940s, in part under the impact of QFT, to which he made important contributions, including the theory of nuclear forces in the 1930s and especially S-matrix theory in the 1940s. I will not be able to address many steps in his thinking leading him to QM, and it is impossible to consider, or even to know, all these steps. It takes a whole volume (Volume 2) of Mehra and Rechenberg’s mammoth history, and then nearly another volume (Volume 3) to discuss (still not completely) with matrix mechanics as developed by Born, Jordan, and Heisenberg, whose articles total over one hundred pages [20] (v. 2–3) [21,34]. A new translation and instructive commentary are offered in [35]. I shall only discuss the key steps of Heisenberg’s invention of QM, manifesting the creative force of mathematics in it, in his original paper [36]. As noted from the outset, focusing on concepts and their history allows one to negotiate the historical complexities involved by avoiding definitive claims concerning the exact time of Heisenberg’s invention of his concepts or their exact sequence. It is still not commonly known and to some surprising that Heisenberg invented QM by considering a toy model of an anharmonic quantum oscillator, rather than the hydrogen atom, the ultimate target of his theory at the time, and hopefully extendable beyond it [36]. In fact, however, doing so helped his discovery by allowing him to perceive some key possibilities for his new mathematics that would have been obscured if he had considered the hydrogen atom, a more complex system [20] (v. 2, pp. 231–233). QM was given its full-fledged (Hamiltonian) form, as matrix mechanics, by Born, Jordan, and Heisenberg himself [21,34]. It was also given, independently, a more abstract form by Dirac [37] and then, in 1926, that of wave mechanics by Schrödinger [38].

As Heisenberg explained at the outset of his paper, the old quantum theory tried to emulate classical mechanics by using the same physical quantities, some of which, such as the period of orbital revolution of the electron, were no longer observable, to predict observable quantities, such as the energy levels of the hydrogen atom. As noted, the approach, while it had major successes, remained incomplete and ultimately proved to be “unfeasible.” These difficulties compelled Heisenberg to adopt a different strategy, also by reversing this approach, which retained the variables of classical mechanics while ad hoc changing, by using Bohr’s correspondence principle, classical equations to achieve better predictions. Heisenberg, by contrast, adopted classical equations unchanged, while introducing new variables. This was both innovative and unexpected, as nobody else thought in this way at the time. So was the change in the role of these equations, as no longer representing the motion of electrons but only used for probabilistic predictions of effects of the interaction between quantum objects (in this case, as Heisenberg considered spectra, photons emitted by electrons) and measuring instruments. The correspondence principle was made by Heisenberg into a mathematical postulate as well. As such, it was one of the motivations for his decision to retain the equations of classical mechanics. In the classical limit, as for large quantum numbers, when electrons are far from nuclei, one could, as in using the original correspondence principle, assume Planck’s *h* to be equal to zero and treat electrons as orbiting nuclei by the laws of classical mechanics and radiating by the laws of classical electrodynamics. Quantum variables convert into classical variables. The behavior of electrons there is still quantum and can have quantum effects, although their probability is low [3] (v. 1, p. 18) [8] (pp. 31–32) [39] (pp. 73–76).

Heisenberg began his derivation of QM with an observation that reflected a radical departure from all preceding physics, including the old quantum theory: “in quantum theory it has not been possible to associate the electron with a point in space, *considered as a function of time*, by means of observable quantities. However, even in quantum theory it is possible to ascribe to an electron the emission of radiation” [36] (p. 263; emphasis added). This emission leads to a quantum jump of the electron. By referring to what happened between experiments, via the concept of emission (even assuming that such an emission cannot be observed as such), this statement is in conflict with both the RWR and Heisenberg’s materialist Platonist view. Either implies the impossibility of referring by language or even physical concepts, such as emission, to what happens between observations [1] (pp. 178–179). These considerations were, however, to come later. A measurement can associate an electron with a point in space. It is, however, not possible to do so by linking this association to a (real) function representing the continuous motion of the electron in space and time. Heisenberg says next [36] (p. 263): “In order to characterize this radiation we first need the frequencies which appear as functions of two variables. In quantum theory, these functions are

*v*(*n*, *n* − *α*) = 1/*h* {*W*(*n*) − *W*(*n* − *α*)}

and in classical theory in the form

*v*(*n*,*α*) = *αv*(*n*) = *α*/*h*(*dW*/*dn*)”.

These formulas, adopted from Bohr’s 1913 theory [12], lead to the difference between classical and quantum phenomena as regards the combination relations for frequencies, which, in the quantum case, correspond to the Rydberg–Ritz combination rules. These rules reflected “the discrepancy between the calculated orbital frequency of the electrons and the frequency of the emitted radiation” [36] (p. 263). Assuming this discrepancy as a postulate, incompatible with classical electrodynamics, was yet another radical feature of Bohr’s 1913 theory [12], discussed below. However, as Heisenberg noted next, “in order to complete the description of radiation [in accord with the Fourier representation] it is necessary to have not only frequencies but also the amplitudes” [36] (p. 263). Heisenberg’s decision to use the Fourier representation in his formally classical equations proved to be crucial for developing his formalism, including as concerns its noncommutative characters. Hendrik Kramers used the Fourier representation in considering dispersion in the old quantum theory, an approach followed in [40], in which noncommutativity was implicit, but remained unnoticed [20] (v. 2, pp. 224–225) The key difference, defining Heisenberg’s most revolutionary mathematical contribution, was in his new variables, which were expressly noncommutative, an entirely new mathematical feature in physics. It is true that relativity previously used tensors of the second rank, which linearly map vectors to vectors and whose components transform according to a specific rule under a change of basis and (while more general objects) can be represented as matrices, in general do not commute. This noncommutativity, however, does not play the same role in relativity, and it did not attract attention. It is only with QM that noncommutative mathematics becomes used in physics in an essential way. The equations of QM must, thus, formally contain amplitudes as well as frequencies. Variables representing these amplitudes could not, however, be the same, because they would not give correct predictions for all quantum numbers. Because Heisenberg’s variables were no longer part of a continuous representation of motion, the “amplitudes” associated with them were no longer amplitudes of physical motions, as classical amplitudes were, making the term amplitude “symbolic” [3] (v. 1, p. 11). They were formal mathematical entities, defined over C, making linear superposition, crucial to QM, purely mathematical and thus symbolic as well. They were, in effect, “probability (density) amplitudes,” linked, via Born’s rule, to the probabilities of discrete transitions between stationary states, corresponding to electrons’ energy levels (with Bohr’s orbits abandoned, as unobservable), manifested in the spectral data observed in quantum experiments. In terms of Hilbert-space formalism, the probability amplitude is $\left\langle \lambda_{i}|\psi \right\rangle$ ($\lambda_{i}$ is an eigenvalue and ψ is the wave function). Born’s rule says that the corresponding probability is the square of the amplitude or the amplitude multiplied by its complex conjugate, or P_i_ = ${\left\langle \lambda_{i}|\psi \right\rangle }^{2}$. Heisenberg used a form of Born’s rule for the transitions between stationary states. Heisenberg then argued as follows:

The amplitudes may be treated as complex vectors, each determined by six independent components, and they determine both the polarization and the phase. As the amplitudes are also functions of the two variables *n* and α, the corresponding part of the radiation is given by the following expressions [36] (p. 263):

 Quantum-theoretical:

Re{*A* (*n*, *n* − *α*)e*^iω^*^(*n*,*n*−*α*)*t*^}

 Classical:

Re{*A_α_* (*n*)e*^iω^*^(*n*)*αt*^}.

The following difficult and, “at first sight,” nearly insurmountable problem now arises: “The phase contained in *A* would seem to be devoid of physical significance in quantum theory, since frequencies are in general not commensurable with their harmonics” and, as a result, “a geometrical interpretation of such quantum-theoretical phase relations in analogy with those of classical theory seems at present scarcely possible” [36] (pp. 263–265). The incommensurability of frequencies with their harmonics, incompatible with classical electrodynamics, was one of the most radical and audacious features of Bohr’s 1913 theory [12]. Bohr *dissociated* the frequency of the radiation emitted by an electron from the frequency of the electron’s orbiting the atom by assuming the energy of the emission to be $E=\frac{1}{2}nhv$. This discrepancy in fact disappears in Heisenberg’s theory because, in contrast to the frequencies of radiation, the orbital frequencies are not observable and, in the absence of orbits, are even no longer meaningful. Heisenberg is, however, ready to solve this problem. As he says: “However, we shall see presently that also in quantum theory the phase has a definitive significance which is *analogous* to its significance in classical theory” [36] (p. 264; emphasis added). “Analogous” only means that the way in which the phase enters mathematically in quantum theory is analogous to the way it enters mathematically in classical theory. Because Heisenberg only considered an anharmonic quantum oscillator, all he needed was a Newtonian equation for it, rather than the Hamiltonian ones required for a full-fledged QM. His thinking appears to have been: My toy model comes short of a full-fledged QM, but that this model works suggests that my conception of the formalism should enable one to develop QM in the same way. This proved to be the case. He says [36] (p. 264): “a given quantity *x*(*t*) [a coordinate as a function of time] in classical theory, this can be regarded as represented by a set of quantities of the form

*A*_*α*_ (*n*)e^*iω*(*n*)*αt*^,

which, depending on whether the motion is periodic or not, can be combined into a sum or integral which represents *x*(*t*):

$$x\left(n,t\right)=\sum_{-\infty }^{+\infty }\alpha A_{n}\left(n\right)e^{i\omega (n)\alpha t}$$

or

$$x\left(n,t\right)=\int_{-\infty }^{+\infty }A_{\alpha }(n)e^{i\omega (n)\alpha t}d\alpha ”.$$

Heisenberg next makes his most decisive and extraordinary move. He notes that “a similar combination of the corresponding quantum-theoretical quantities seems to be impossible in a unique manner and therefore not meaningful, in view of the equal weight of the variables *n* and *n* − *α*.” However, he says, “one might readily regard the ensemble of quantities *A* (*n*, *n* − *α*)e*^iω^*^(*n*,*n*−*α*)*t*^ as a representation of the quantity *x*(*t*)” [36] (p. 264). Heisenberg, again, did not think about these ensembles as matrices, an equivalence only realized by Born. It was a great innovation, little prepared by the preceding history of physics. It was given no derivation from first principles either. These variables reflected the fact that, as he said later, “the equations of [QM] form themselves *postulates* of the theory. … [T]heir ultimate justification lies in the agreement of their predictions with the experiment” [8] (p. 108). Thus, the classical function *x*(*t*), over R, representing a motion of the electron, is replaced with an operator, in effect, in a Hilbert space over C.

Another way of achieving the same outcome would be by introducing, as Schrödinger did in his time-dependent equation, the ψ-function, as a Hilbert space vector, with “observables” (mathematical entities linked to physical observations), such as the position, momentum, or energy, *H*, becoming operators:

$$i\hbar \frac{d}{dt}\;\left|\left.\psi \left(t\right)\right\rangle =H\right|\left.\psi \left(t\right)\right\rangle .$$

The mathematical equivalence of both schemes is nearly automatic, and was quickly realized, although it was only rigorously established by von Neumann [41]. Most formally, this equivalence follows from the Stone–von Neumann theorem, establishing the uniqueness of the canonical commutation relation, pq−qp=iℏ, for position and momentum operators, defining the Lie algebra of QM for continuous variables, requiring infinite-dimensional Hilbert spaces.

Back to Heisenberg’s original argument, another problem arises, however, once these ensemble variables are in place. This problem was solved long before then in matrix algebra, but Heisenberg did not know this. These ensembles themselves do not establish an *algebra*, for which one needs rules for adding and multiplying them. Otherwise, one could not use them in equations. For his toy model, Heisenberg only needed the square of the coordinate variable, *x*(*t*)^2^, which does not involve noncommutativity. Heisenberg proposed, again, without any physical justifications [36] (p. 265): “it seems that the simplest and most natural assumption would be to replace classical [Fourier] equations … by

$$B\left(n,n-\beta \right)e^{i\omega \left(n,n-\beta \right)t}=\sum_{-\infty }^{+\infty }\alpha A\left(n,n-\alpha \right)A(n-\alpha ,n-\beta )e^{i\omega \left(n,n-\beta \right)t}$$

or

$$\int_{-\infty }^{+\infty }A\;\left(n,n-\alpha \right)A\left(n-\alpha ,n-\beta \right)e^{i\omega \left(n,n-\beta \right)t}d\alpha .”$$

This is the main algebraic postulate, the multiplication postulate, of Heisenberg’s theory, “an almost necessary consequence of the frequency combination rules” [36] (p. 265). The word “almost” indicates that it is more invented, almost guessed, than derived. Although it is commutative in the case of *x*^2^, this multiplication is in general noncommutative, expressly for position and momentum variables, *PQ* − *QP* ≠ 0, as Heisenberg knew. His toy model allowed him to avoid dealing with mathematically more complex noncommutative multiplication. Without realizing this, however, he used this noncommutativity in solving his equation, as Dirac was the first to notice [20] (v. 4, p. 129). This feature proved to be momentous physically. Most famously, it came to represent the uncertainty relations, in the formalism of QM and the mutually exclusive or complementary character of the measurements defining them, which required these variables to be infinite-dimensional and unbounded. This noncommutativity was, initially, off-putting to some, including Heisenberg himself and Pauli, although not to Born or Dirac. Dirac quickly realized its importance and made it the starting point of his mathematically driven derivation of QM, by using the quantum analogue of the Poisson bracket, in fact, an element of the Lie algebra of the theory. He derived the first “official” equation of the full-fledged QM: $pq-qp\;=\;\frac{h}{2\pi }I$ (**I** is the identity matrix), independently of Born and Jordan [20] (v. 3, p. 69) [27] (p. 862) [37]. (he appearance of *h*, which did not figure in Heisenberg’s derivation considered thus far, is necessary in all quantum equations used for making actual predictions. Born and Jordan, and Dirac also defined, algebraically, in the style of Leibniz’s calculus, “symbolic differentiation,” enabling one to retain the differential equations of classical mechanics and their accompanying machinery, such as the Poisson bracket, while using new quantum variables, as Hilbert space operators. Dirac’s starting point, again, in the style of Leibniz, was the quantum-mechanical analogue of the rule for the differential, *d*, of the product of two functions, $d\;\left(f\cdot g\right)=g\cdot df+f\cdot dg,$ which may be seen as a linear operator and suitably quantized [37]. As was soon realized [42], one can associate unitary representations of Lie groups with Hilbert spaces of quantum systems in QM, just as one can associate them with symplectic manifolds mapping, representationally, classical Hamiltonian systems, dealing with functions over R. In both classical and quantum mechanics, Poisson brackets are associated with Lie algebras of these Lie groups.

In effect, however, all the key features of the formalism of QM, axiomatized by von-Neumann’s Hilbert-space version, were contained in Heisenberg’s paper, apart from Schrödinger’s equation, used as an axiom, rather than derived, by von Neumann. The term “Hilbert space” was introduced by von Neumann following QM and under its impact, because his article, where the term first appeared, considered unbounded operators, which, as noted, were not studied in mathematics before QM [43]. Heisenberg spoke of his new algebra of matrices as the “new kinematics,” which, as explained above, was misleading because his new variables were not related to motion. In Einstein’s view, the theory was not even a mechanics: it was not a representation of the physical dynamics of individual systems, but only predicted, probabilistically, what is observed in measuring instruments. This assessment may, however, depend on how one understands mechanics. Heisenberg’s kinematics was the *mathematics* of transition probabilities between quantum phenomena observed in measuring experiments.

Heisenberg’s approach, driven by the creative force of mathematics, made QM engendered by an abstract mathematical scheme. This type of approach, conceived still more formally, was used by Dirac in creating his version of QM and then quantum electrodynamics (QED). Later, Dirac advocated this approach as “the most powerful method of advance” in quantum theory:

The most powerful method of advance that can be suggested at present is to employ all the resources of pure mathematics in attempts to perfect and generalise the mathematical formalism that forms the existing basis of theoretical physics, and after each success in this direction, to try to interpret the new mathematical features in terms of physical entities.

[15] (p. 1)

The greatest innovations, such as those of Heisenberg and Dirac, come, however, when using the resources of pure mathematics *transforms* “the mathematical formalism that forms the existing basis of theoretical physics,” in other words, when this generalization changes “the existing basis of theoretical physics.” Dirac’s 1931 [15] statement was made in response to the problems of QED, eventually solved by renormalization around 1950, without replacing the existing formalism with a new one, but rather by working, sometimes innovatively, with the existing formalism. This solution was never accepted by Dirac and has been and remains unsatisfactory to others. Dirac, however, used this method beginning with his work on QM and QED. So did Heisenberg in creating QM, becoming the inventor of this method in its transformative character. Heisenberg’s original paper served as an inspiration for Dirac, especially in using noncommutative mathematics, which initially worried Heisenberg but proved so fruitful for Dirac and Heisenberg himself in his subsequent work. As indicated earlier, however, while, as that of Dirac, Heisenberg’s thinking was driven by the creative force of mathematics, the impetus for this thinking, unlike that of Dirac, was the nature of quantum phenomena, even if without representing, at least physically, the ultimate reality responsible for them.

## 4. Physics as Mathematics, Mathematics as Physics: Heisenberg’s Materialist Platonism

This section considers Heisenberg’s materialist Platonism, with a broader aim of offering a critique of Platonism in general, keeping in mind that, as stated from the outset, this critique is not a dismissal of Platonism but rather resituating and redelimiting it. Heisenberg’s move from the RWR view towards his materialist Platonism was not surprising. Heisenberg always, including at the time of his invention of QM, accompanied by his reading of Plato’s *Timaeus*, had affinities with Plato’s thought. I define his Platonism as *materialist* because Heisenberg maintained that the mathematics of quantum theory represented a material physical reality. He also assumed the Bohr postulate, stating that quantum phenomena, along with the observable parts of measuring instruments, are represented by classical physical concepts. Doing so positions us and our own (material and mental) reality in relation to quantum phenomena and their (classical) reality. This positioning itself is not different from our positioning as observers or theorists in classical physics or relativity. The difference is in the character of quantum phenomena and the way we can or cannot technologically observe and theoretically, specifically mathematically, predict the data contained in them. Most forms of Platonism concern mental reality, and some, such as Plato’s own, deny the existence of material reality. On the other hand, Heisenberg’s materialist Platonism was different from most realist views of fundamental physics, based on mathematized *physical* concepts in dealing with the ultimate reality considered, a view always rejected by Heisenberg, beginning with his invention of QM, just as it would be in the RWR view. The latter, however, also rejects a purely mathematical representation or conception of the ultimate reality responsible for quantum phenomena.

It is difficult to ascertain when materialist Platonism came to expressly define Heisenberg’s view. Some intimations of it could be discerned in his Göttingen lectures of 1926 [44], his 1927 uncertainty relations paper [39], and especially his Chicago lectures of 1929, which contained parallels with later works, governed by materialist Platonism [8]. By the late 1930s, Heisenberg’s views were also influenced by the developments of QFT, especially the role of symmetries there, with which elementary particles became associated by linking them to irreducible representations of symmetry groups, as discussed below. Another key factor was the problem of the infinities in QED and then in QFT for weak and strong forces (QED was renormalized by 1950). Heisenberg’s work on the S-matrix in the 1940s was connected with this problem. Eventually, Heisenberg envisioned a new concept, the *Weltformel* [World-formula], a nonlinear spinor equation, that would represent the ultimate physical reality as defined by all fundamental forces of nature. No viable equation of this type was ever found by Heisenberg, and QFT developed differently from the way Heisenberg envisioned. It was the Yang–Mills, a nonabelian gauge QFT, introduced around the same time, that proved to provide the basis for elementary particle physics, leading to the Standard Model [45]. Pauli sketched a theory similar to that of Yang–Mills a bit earlier, but did not publish, discouraged by the problem of the massless excitations in the theory, only solved in the 1960s by the symmetry-breaking mechanism. Chen Ning Yang and Robert Mills admitted this problem in their article [45] (p. 195) but were bold enough to publish their theory, nonetheless.

An intriguing question may be whether, even at the time of his invention of QM, while assuming, just as Bohr did, that QM “does not deal with a space–time description of the motion of atomic particles” [3] (v. 1, p. 48), Heisenberg already entertained something akin to materialist Platonism, rather than closer to Bohr’s RWR view (then the weak one) assumed at the time of this statement. This statement allows for a materialist Platonism because the latter does not deal with this description, which is physical, either. Heisenberg’s paper introducing QM may suggest the RWR view by saying “a geometrical interpretation of such quantum-theoretical phase relations in analogy with those of classical theory seems at present scarcely possible” [36] (pp. 263–265). However, because this particular geometrical interpretation is associated with a physical concept, it is possible that these relations are part of a purely algebraic or abstract geometrical representation of the reality responsible for quantum phenomena. Heisenberg’s articles and correspondence at the time do not appear to reflect on this subject. His 1929 comments, in his Chicago lectures, may, however, be seen as gesturing toward materialist Platonism:

It is not surprising that our language [and concepts] should be incapable of describing processes occurring within atoms, for ... it was invented to describe the experiences of daily life, and these consist only of processes involving exceedingly large numbers of atoms. Furthermore, it is very difficult to modify our language so that it will be able to describe these atomic processes, for words can only describe things of which we can form mental pictures, and this ability, too, is a result of daily experience. Fortunately, *mathematics is not subject to this limitation*, and it has been possible *to invent a mathematical scheme*—the quantum theory [QM]—which seems entirely adequate for the treatment of atomic processes.

[8] (p. 11; emphasis added)

I add concepts (in their everyday sense) because language is indissociable from concepts, which mediate the relationships between words and things, and give meaning to words. Heisenberg does so himself earlier in the book, even by identifying ordinary language and classical concepts “ordinary language (i.e., classical concepts)” [8] (p. 2). Quantum objects, such as Josephson devices, could be macroscopic, but their quantum nature is defined by their microscopic quantum constitution, which would preclude their description by means of ordinary language or concepts, as Heisenberg says. Words can also describe things of which we cannot form mental pictures. These qualifications, however, do not undermine Heisenberg’s point, which amounts to the fact that our language and thinking are the product of our evolutionary biological constitution defined by the experiences dealing with objects consisting of “exceedingly huge numbers of atoms.” There is, accordingly, no special reason to assume that our thinking and language will be able to describe nature on quantum scales, or conversely, on very large, cosmological scales. Instead, one might doubt that one can do so even mathematically. If mathematics, too, is only human and is the product of the same evolutionary development, why would it be able to represent or even deal otherwise, beyond certain limits, with a reality that is not human? We are indeed fortunate to be able to have and to use mathematics to predict quantum phenomena. In fact, we are fortunate to be able to use mathematics in all our physics thus far, because the freedom of mathematics from the limitations of common language and concepts does not guarantee it will work beyond its present-day limits. QFT might not work beyond the scale of the Standard Model, 10^2^ GeV. The grand unification theory (GUT) scale (for all fundamental forces, apart from gravity), a theory that does not yet exist and may not be found, is 10^16^ GeV, still below the Planck scale, 10^19^ GeV.

There is still the question in what sense the “mathematical scheme” of QM is “*entirely* adequate for the treatment of atomic processes,” whether by this “adequacy” Heisenberg refers to the weak RWR view or something akin to his materialist Platonist view. Both views preclude applying physical concepts or even (they may not be concepts) the ideas of space and time to this reality, vs. quantum phenomena. This is different from considering space and time as emerging forms of physical reality, as in some recent approaches, or rather the latter would pose the same question concerning the reality, possibly an RWR one, underlying that of time and space, or of another actual reality observed, possibly only by our instruments. The situation is closer to Kant’s epistemology of phenomena, as representations in our thought, vs. things-in-themselves, which are beyond such representation, but as noted, the RWR case is more radical. Although Kant places things-in-themselves beyond knowledge, he allows that one can form a conception of them rather than, as in the strong RWR view, placing them beyond conception, even to the point of being neither material nor mental, not an idea found in Kant. It may be shown that even Kant’s view of things-in-themselves as beyond knowledge remains a form of realism rather than the weak RWR view [2] (pp. 58–59). On the other hand, while Heisenberg’s materialist Platonism is closer to Kant insofar as “things-in-themselves” are at least conceivable or even representable, they, as against Kant, become mathematical for Heisenberg. As he said, “Kant’s ‘thing-in-itself’ is for the atomic physicist, if he uses this concept at all, finally a mathematical structure.” Heisenberg added, however, thus making his Platonism materialist: “But this structure is—contrary to Kant—indirectly deduced from experience,” defined by quantum experiments [1] (p. 83). Not everyone, beginning with Einstein, would have seen either the RWR view (especially disconcerting to Einstein) or materialist Platonism as “*entirely* adequate.” Einstein and his followers wanted realism defined by physical concepts, thus requiring new physical concepts in quantum theory. Einstein’s appeal to such new concepts was shared by Schrödinger and countered by Bohr from the RWR perspective, coupled to the Bohr postulate [22] (pp. 26–34) [46] (v. 7, pp. 505–511).

In his later works, Heisenberg maintained the same position as concerned the incapacity of general language and concepts to represent the ultimate reality responsible for quantum phenomena, but now expressly assuming materialist Platonism. As he said:

There is no description of what happens to the system between the initial observation and the next measurement. … Any kind of understanding, scientific or not, depends on our language, on the communication of ideas. Every description of phenomena, of experiments and their results, rests upon language as the only means of communication. The words of this language represent the concepts of daily life, which in the scientific language of physics may be refined to the concepts of classical physics. These concepts are the only tools for an unambiguous communication about events, about the setting up of experiments and about their results. If therefore the atomic physicist is asked to give a description of what really happens in his experiments, the words ‘description’ and ‘really’ and ‘happens’ can only refer to the concepts of daily life or of classical physics. As soon as the physicist gave up this basis, he would lose the means of unambiguous communication and could not continue in his science. Therefore, any statement about what has ‘actually happened’ is a statement in terms of the classical concepts and—because of thermodynamics and of the uncertainty relations—by its very nature incomplete with respect to the details of the atomic events involved. The demand to ‘describe what happens’ in the quantum-theoretical process between two successive observations is a contradiction in adjecto, since the word ‘describe’ refers to the use of the classical concepts, while these concepts cannot be applied in the space between the observations; they can only be applied at the points of observation. … [T]he problems of language are really serious. We wish to speak in some way about the structure of the atoms and not only about ‘facts’—the latter being, for instance, the black spots on a photographic plate or the water droplets in a cloud chamber. However, we cannot speak about the atoms in ordinary language [or in terms of ordinary concepts].

[1] (pp. 47, 145, 178–179)

This reasoning, again, allows for either the RWR view or materialist Platonism. It is humanly natural to assume that something happens between observations, given the changes that we observed in the physical states of the instruments used. The sense that something happened is one of the most essential aspects of human experience. However, in either the RWR view or materialist Platonism, the expression “something happened” is inapplicable to the ultimate reality responsible for quantum phenomena. It is, in principle, possible to assume that, while mental, some mathematics would *represent* this kind of reality. This possibility is precluded by the RWR view because this assumption would imply assigning this reality a structure, and hence assuming at least a conception of this reality, disallowed by the RWR view. Materialist Platonism cannot exclude the possibility of the existence of the ultimate RWR reality beneath that represented by a materialist Platonist theory or interpretation. In this case, however, materialist Platonism is no longer applicable in considering this reality, which would instead conform to the RWR view. While Heisenberg might have agreed with this point, there is no evidence that he ever considered it. Both Bohr, in adopting the RWR view, and Heisenberg, in adopting his *materialist* Platonism, assumed this reality to be material. On the other hand, while for both “one would get into hopeless difficulties if one tried to describe what happens between two consecutive observations” [1] (p. 52), only Heisenberg’s materialist Platonist view, and not that of Bohr, as the RWR view, allows one to represent the reality between such observations purely mathematically. This possibility is defined by the dependence of all (rather than only classical) physical concepts on ordinary language and concepts, while a purely mathematical representation is free from this dependence. This logic leads Heisenberg to his (materialist) Platonism, virtually by name:

For the sake of comparison with modern atomic physics it is important to mention the explanation of matter given by Plato in his dialogue *Timaeus*. Plato was not an atomist; on the contrary, Diogenes Laertius reported that Plato disliked Democritus so much that he wished all his books to be burned. But Plato combined ideas that were near to atomism with the doctrines of the Pythagorean school and the teachings of Empedocles. The Pythagoreans seem to have been the first to realize the creative force inherent in mathematical formulations. … There was also much mysticism in the doctrines of the Pythagorean school which for us is difficult to understand. But by making mathematics a part of their religion they touched an essential point in the development of human thought.

[1] (64–65)

While strictly maintaining the difference between ancient Greek thought and modern science, Heisenberg always saw QM and QFT as a continuation of this line of thought, as defined by “the creative force inherent in mathematical formulations.” *Timaeus* provides a link between his later thinking and that leading him to his discovery of QM. Heisenberg was reading *Timaeus* at the time, including in Helgoland, making this reading part of the Helgoland legend. Hence, as I suggest here, it is possible that materialist Platonism was part of Heisenberg’s “quantum unconscious” all along, which, if true, gives a new perspective on his discovery of QM and the creative force of mathematics in this discovery.

One can easily see Heisenberg’s reasoning for moving from the impossibility of “speak[ing] about the atoms in ordinary language” to the impossibility of physical concepts, the ultimate reality responsible for quantum phenomena. Physical concepts, even when mathematized, can never be entirely freed from language and concepts expressed in language. Mathematics is, by contrast, in principle free from this limitation. The question is, however, whether the mathematics of QM or QFT, or any mathematics, can represent the ultimate reality responsible for quantum phenomena. This claim is not falsifiable, given that in Heisenberg’s own account, this behavior, now given a mathematical ontology, is *unobservable*. Similar problems would arise in the case of ontic structural realism, which is, as noted, close to Heisenberg’s materialist Platonism in this respect, although not mathematically. What is falsifiable, and has been verified, is that the mathematics, QM or QFT, correctly predicts the outcomes of all quantum experiments thus far. QED is the best confirmed physical theory ever. It is true that the assumption of RWR-type reality is not falsifiable either. It offers, however, no ontology of the ultimate reality responsible for quantum phenomena, which is a crucial difference. Moreover, while presumably purely mathematical, Heisenberg’s ontology is grounded in an Aristotelian concept of potentiality, which may not be possible to define purely mathematically. Heisenberg notes first:

A real difficulty in the understanding of this [materialist Platonist] interpretation arises, however, when one asks the famous question: But what happens ‘really’ in an atomic event? It has been said before that the mechanism and the results of an observation can always be stated in terms of the classical concepts. But what one deduces from an observation is a probability [wave] function, a mathematical expression that combines statements about possibilities or tendencies with statements about our knowledge of facts. So we cannot completely objectify the result of an observation, we cannot describe what ‘happens’ between this observation and the next.

[1] (p. 50)

This statement, as such, allows for either a materialist Platonist or RWR view. In the RWR view, a wave function only enables expectations concerning possible future experiments on the basis of previously performed experiments, in the absence of any ontology, vs. Heisenberg’s ontology of potentiality, which is difficult to justify. As he says in closing his argument concerning his interpretation, on this point different from that of Bohr, to whom it is indebted in other respects (which explains why Heisenberg refers to his interpretation as the Copenhagen interpretation, introducing the term itself):

All these difficult definitions and distinctions can be avoided if one confines the language to the description of facts, i.e., experimental results. However, if one wishes to speak about the atomic particles themselves one must either use the mathematical scheme as the only supplement to natural language or one must combine it with a language that makes use of a modified logic or of no well-defined logic at all. In the experiments about atomic events, we have to do with things and facts, with phenomena that are just as real as any phenomena in daily life. But the atoms or the elementary particles themselves are not as real; they form a world of potentialities or possibilities rather than one of things or facts.

[1] (p. 159)

The difficulty is the expression “not as real.” If “*the elementary particles themselves are not as real*” as observed quantum phenomena, how real are they and in what sense? This question remains in place even assuming that they can be represented purely mathematically. Given that they are no longer things, not even Kant’s things-in-themselves, but only things-in-themselves as “mathematical structure[s]” [1] (p. 91), in what sense do they form a world of potentialities or possibilities? For whom or, if this world is not human, for which entities, is his world of potentialities formed? In RWR interpretations, the ultimate *reality* responsible for quantum phenomena is, while inconceivable, *real* as something that exists apart from us. On the other hand, our interactions with this reality by means of experimental technology and thought, including mathematics, do create a world of new possibilities, defined by our experiments. These possibilities may be seen as potentialities insofar as they may exclude certain other possibilities, in view of Bohr’s complementarity, for example. Of course, in predicting these possibilities, which can only be done by means of the mathematics of QM or some other theory, “speaking about the atomic particles themselves one must … use the mathematical scheme as the only supplement to natural language” capable of doing so. The superposition of mathematical state vectors in the formalism enabling predictions concerning future events is clearly on Heisenberg’s mind here. It does not follow, however, that atomic particles themselves “form a world of potentialities or possibilities.” Such potentialities or possibilities only exist for us in our human world, to which, in the present view, all mathematics belongs as well. It is real as part of our mental world and, along with physics, of our culture. There is no world of potentialities or possibilities otherwise. See, however, ref. [47] for a realist view of potentiality, which does not consider Heisenberg’s materialist Platonism. Quantum experiments do change the present state of reality and define, by quantum causality, a possible course of reality, but only because of human intervention by means of experimental technology.

Accordingly, the only “potentiality” one can speak of is that defined by the fact that each quantum experiment defines a set of possible future experiments and their possible numerical outcomes. If one cannot speak of what happens between quantum experiments, can one say that the “world” of elementary particles is the world of potentialities, as an independent world? It may be seen as such for us, which is perhaps what Heisenberg means, but this is not what he says. It is also possible that what happens between quantum experiments can only be described by the mathematics of quantum theory, in the absence of physical concepts, while if we want to use human language, we can only speak of potentiality. Heisenberg does not say this either, however. Some *nonmathematical* sense of potentiality in considering what happens between experiments appears to be assumed, in conflict with materialist Platonism.

What, then, of elementary particles? In his materialist Platonist phase, Heisenberg argued that the physical concept of a fundamental particle should be replaced by the mathematical concept of a fundamental symmetry. He saw this necessity as a consequence of Dirac’s discovery of antimatter, “perhaps the biggest change of all the big changes in physics of [the twentieth] century” because “one of the most spectacular consequences of Dirac’s discovery [was] that the old concept of the elementary particle collapsed completely” [48] (p. 31). The reason, as explained, is that in high-energy quantum regimes, one may not observe the particle of the same type, say, an electron, in two successive experiments, because the second one can register as a positron to the next. This situation is fully consistent with RWR interpretations [2] (pp. 273–306). According to Heisenberg, however:

What then has to replace the concept of a fundamental particle? I think we have to replace this concept by the concept of a fundamental symmetry. The fundamental symmetries define the underlying law which determines the spectrum of elementary particles. … [W]hat we have to look for are not fundamental particles, but fundamental symmetries. And we have actually made this decisive change in the concepts, which came about by Dirac’s discovery of antimatter, that I do not think we need any further breakthrough to understand the elementary—or rather nonelementary—particles. We must learn to work with this new and unfortunately rather abstract concept of the fundamental symmetries; but this may come in time.

[48] (p. 36)

These symmetries are irreducible representations of symmetry groups, Lie groups, the concept introduced by Eugene Wigner [49]. Importantly, consistently with the RWR view, these representations are infinite-dimensional ones in Hilbert spaces over C and not in the physical three-dimensional space, defined over R, where one observes quantum phenomena, which exhibit their own symmetries, such as those associated with conservation laws by Noether’s theorems. Quantum theory had been using this concept for decades before Heisenberg’s statement. His point is a *replacement* by this concept of that of the elementary particle, in accord with his mathematical Platonism.

## 5. Quantum Symmetries, from Galois and Heisenberg and from Heisenberg to Galois

This section reconsiders the role of symmetries and group theory in QM and, especially, QFT. This role was, as just explained, strongly advocated by Heisenberg as part of his materialist Platonism, even to the point of replacing the concept of fundamental particles with that, purely mathematical, of fundamental symmetries. The present argument gives symmetries and group theory equally important roles in QM and QFT, while, however, assuming the RWR view of this role. It is not surprising that Heisenberg, in his later works, turns to the concept of elementary or (they are one and the same) fundamental particles. By 1930, QFT became most fundamentally an elementary particle theory and vice versa. While terms are sometimes used separately or differently, the elementary particle theory is QFT, technically a set of QFTs. Admittedly, as the name “quantum field theory” suggests, these are theories of quantum fields. This concept, however, continues to be accompanied by that of the elementary particle, even if sometimes in order to dispense with the latter. The title “What is an elementary particle?” has been used by several major figures, including by Heisenberg himself, in supporting the replacement of the concept of elementary particles with that of fundamental symmetries [48] (pp. 81–88), Schrödinger, around the same time, while in returning to his early idea of quantum waves [50], and Stephen Weinberg, with an emphasis on quantum fields [51].

Virtually all concepts of the elementary particle in QFT, or QM, contain the following feature, special to quantum physics: while elementary particles of the same type cannot be distinguished from each other, except in some experiments in which more than one particle is involved, these types themselves are rigorously distinguishable, and all of them known thus far are classified. In any quantum regime, say, two electrons could be distinguished by changeable properties associated with them, such as their positions in space or time, momenta, energy, or the directions of spins. Such properties are subject to the uncertainty relations and complementarity. It is possible to locate two different electrons in separate regions in space, or, by the Pauli exclusion principle, in different states, unlike photons or other bosons (particles with an integer spin), two or more of which could be in the same state. On the other hand, it is not possible to distinguish two electrons or any two particles of the same type by their mass, charge, or spin. These quantities are not subject to the uncertainty relations or complementarity. In RWR interpretations, properties defining elementary particles within each type could only be *associated* with them by means of the effects observed in measuring instruments and are not attributable to quantum objects themselves, even at the time of measurement. This, however, allows one to maintain both the indistinguishability of particles of the same type and the distinguishability of these types because both features can be defined by such effects.

In the present interpretation, the *concept* of an elementary particle or any quantum object is only applicable at the time of observation, by the Dirac postulate. Hence, rigorously, one cannot speak of the same quantum object, such as the same electron, in two successive measurements. The assumption that it is the same electron is, however, a statistically permissible idealization in low-energy (QM) regimes. A statistical dimension of the situation remains in place because one cannot be certain that one encounters the same electron after it was emitted from a source, even in low-energy (QM) regimes, although the probability that it would be a different electron is low. As explained below, in high-energy quantum regimes, speaking of the “same” electron as detected in two successive observations loses its meaning altogether. In the present interpretation, speaking of an elementary particle or any quantum object *nearly* amounts to a protocol for establishing the relationships, in general probabilistic, between quantum events—nearly, because the quantum object considered is still assumed to exist, as something beyond conception, at the time of observation. All that is *physically* observed, however, is a property of the observed part of the instrument used and not of the quantum object considered. The elementary character of a particle is defined by the fact that there is no experiment that allows one to associate the corresponding effects on instruments with more elementary individual quantum objects. Once such an experiment becomes conceivable or is performed, the status of a quantum object as an elementary particle could be challenged or disproven, as it happened when hadrons and mesons were discovered to be composed of quarks and gluons. This composite nature will manifest itself in a new set of effects observed in each corresponding experiment. Elementary particles cannot be assumed, on the Democritean model, to be fundamental elementary constituents, “building blocks,” of nature in RWR interpretations, because the latter preclude any assumption concerning this constitution. Nor is it possible to apply to elementary particles any specified concept of particle, any more than any other concept, such as wave or field. As, however, explained below, the concept of quantum field could be defined as a mode of an independent RWR reality, rather than as a quantum object.

As stated, in high-energy quantum regimes, the concept of an elementary particle acquires new features in view of the following situation, to which the mathematical architecture of QFT responds. In fact, with Dirac’s equation, this mathematical architecture was discovered first. Speaking for the moment in classical terms, suppose that one arranges for an emission of an electron, at a given high energy, from a source and then performs an observation at a certain distance from the source by placing a photographic plate. The probability or, if we repeat the experiment with the same initial conditions (defined by the classical state of the emitting device), statistics of the outcomes would be correctly predicted by QED. But what is the outcome? The answer is not what our classical or even quantum-mechanical intuition would expect. This answer was a revolutionary discovery made by Dirac through his equation.

If one considers an electron in the low-energy (QM) regime, it is impossible, because of the uncertainty relations, to predict the place of the collision exactly as is possible in classical physics. The emitted electron could, in principle, be found anywhere in a given area, to which such a prediction applies, or not found at all. Nor can an emission of an electron be guaranteed. There is a small but nonzero probability that a collision will not be observed or that the observed trace is not that of the emitted electron. As noted, however, statistically, one can consider two observed events as related to the same electron, even if one assumes the Dirac postulate. Once, however, one moves to high-energy regimes, beginning with those governed by QED, in subsequent observations, one can find not only an electron (or nothing), but also other particles: a positron, a photon, an electron-positron pair. That is, in RWR interpretations, one can register the events or phenomena that we associate with such entities. It is in responding to this situation that the present interpretation assumes the Dirac postulate. QED predicts which among such events can occur, and with what probability. The corresponding Hilbert-space machinery becomes more complex, in the case of Dirac’s equation, making the wave function a four-component Hilbert-space vector, as opposed to a one-component or, if one considers spin, two-component vector in QM. These features represent the fact that Dirac’s equation is an equation for both the (free) electron and the (free) positron, also containing their spins. They can transform into each other or other particles, such as photons, in the corresponding high-energy processes. In RWR interpretations, these transformations are only manifested in observational instruments, and there is no representation or even conception, physical or mathematical, of what happens between observations.

In the elegant simplicity of its compact symbolic form, reproduced on the plate in Westminster Abbey commemorating Dirac, Dirac’s equation is:

iγ⋅ ∂ψ=mψ.

This simplicity is deceptive, because it encodes a highly complex mathematical machinery. As introduced by Dirac, the equation was:

$$\left(\beta mc^{2}+\;\sum_{k=1}^{3}\alpha_{k}p_{k}c\right)\psi \left(x,t\right)=i\hbar \frac{\partial \psi (x,t)}{\partial t}$$

$$a_{i}^{2}=\beta^{2}=I_{4}$$

(*I*_4_ is the identity matrix)

$$\alpha_{i}\beta +\beta \alpha_{i}=0\;\alpha_{i}\alpha_{j\;}+\alpha_{j}\alpha_{i}=0$$

This equation unfolds into four coupled linear first-order partial differential equations for the four quantities that make up the wave function. ***p*** is the momentum operator. The wave function *ψ*(*t*, ***x***) takes value in a Hilbert space *X* = C^4^ (Dirac’s spinors are elements of *X*). For each *t*, (*t*, ***x***) is an element of *H* = L^2^ (R^3^) ⨂ C^4^. This structure of Dirac’s equation and variables allows one to predict the probabilities of quantum events associated with the relativistic electron, just as Schrödinger’s equation does for the nonrelativistic electron. Schrödinger’s equation is the nonrelativistic limit of Dirac’s equation.

An analogous, if more complex, situation is found in the case of the Yang–Mills equations, introduced as noted in 1953 and developed into Yang–Mills QFT, which governs the Standard Model. In this case, rather than particles, one begins with quantum fields. Mathematically, while technical complexities are formidable, especially in the electroweak theory and QCD, the basic concepts are reasonably well established. Among the standard technical textbooks are [52,53,54]. A comprehensive survey is found in [55]. The physics of quantum fields is, however, a different matter, and it is primarily responsible for the unsettled nature of the concept, arguably even more so than that of the elementary particle. There have been few, if any, essential changes concerning this problem for decades. While there is a broad consensus that a viable, generally assumed, physical concept of a quantum field is necessary, there is no such concept thus far [55]. Most proposed concepts of the quantum field are realist. I shall now sketch the physical concept of the quantum field defined by the strong RWR view, following [2], with some adjustments, in view of this article’s argument. This concept is consistent with the mathematics of QFT and most currently available *mathematical* concepts of quantum field, as based on the Lagrangian formulation and canonical commutation or anticommutation relations for fields, for bosonic and fermionic fields, respectively, analogous to those of QM, say, for the bosonic field, ϕ and φ:

$$\left[\phi \left(x,t\right),\varphi \left(y,t\right)\right]=i\delta^{3}\left(x-y\right)$$

$$\left[\phi \left(x,t\right),\phi \left(y,t\right)\right]=\left[\varphi \left(x,t\right),\varphi \left(y,t\right)\right]=0$$

In this understanding, a quantum field is *not a quantum object* but a particular *mode* of the RWR-type reality, which, as any such mode, is assumed here to exist independently and to be manifested only by its effects on measuring instruments, via quantum objects. A quantum field is independent of measurement, while quantum objects are always defined by measurements, through which they also indirectly define quantum fields. These effects are more numerous than those observed in low-energy regimes. This multiplicity is defined by the fact that these effects correspond to elementary particles, to which a quantum field gives rise, and which can be of various types even in a single experiment, consisting of one or more successive measurements, with the first one performed on a given particle. The initial quantum object could also be a set of elementary particles of the same or different types, with a different such set, possibly consisting of entirely different types of particles, appearing in each new measurement. As a form of the RWR-reality assumed to exist independently, a quantum field is responsible for transforming effects associated with elementary particles created in the process at the time of measurement. These effects may be either invariant (as concerns a given particle type), such as those associated with mass, charge, or spin, or they may be variable, such as those associated with position, momentum, or energy. As concerns this association, always via real (vs. virtual) particles, there is no difference from low-energy regimes; the difference is in the effects observed. These become more numerous in high-energy regimes, with this multiplicity and hence that of the types of elementary particles needed to be considered becoming progressively greater with higher-energy levels.

In this understanding of the concept, speaking, as is common, of the quantum field of a particle, say, an electron, entails new complexities. Mathematically, the formalism of, say, QED, allows one to make predictions concerning the electron, which, mathematically, invites one to speak of the electron as a quantum field. Physically, in the present understanding of a quantum field, this only means that the RWR-type reality defining the quantum field considered has strata that enable the corresponding measurements detecting electrons. It is not possible, however, to separate these strata from those similarly associated with the possibility of detecting a positron or a photon in the same experiment (in the sense of being defined by the same initial measurement as a preparation), because neither of these strata as such is observed in measurement. Only electrons, positrons, or photons are observed, as quantum objects, and only in terms of the corresponding effects in measuring instruments. On the other hand, it is possible to specify quantum fields as associated with fundamental forces and the corresponding types of particles, as field bosons: electromagnetic (photons), weak *W*^+^, *W*^−^, and *Z*, or strong (gluons), all three underlain by the Higgs field and its (Higgs) boson. The role and the concept of virtual particles are put aside here. It was discussed from the present perspective in [2] (pp. 273–306).

The present concept of a quantum field defines a quantum field as part of the independent reality ultimately responsible for quantum phenomena and not as a quantum object, which is, in the present view, only associated with an experiment, rather than assumed to exist independently, by the Dirac postulate. This physical concept of a quantum field can, again, accommodate most currently standard versions of a mathematical concept of a quantum field. The operators enabling one to predict the probabilities for the “annihilation” of some particles and “creation” of others, that is, for the corresponding measurable quantities observed in measuring instruments, annihilation and creation operators, *â* and *â*^†^, each lowering or increasing the number of particles in a given state by one. In RWR-type interpretations, these operators do not represent any physical reality: they only help estimate the probabilities of the outcomes of experiments in high-energy regimes, which QFTs comprising the Standard Model do very well, their technical complexities and yet unsolved problems notwithstanding.

The successes of QFT (beyond QED), culminating in the Standard Model, came from the Yang–Mills 1954 theory and its mathematics, which, eventually, also became part of pure mathematics, specifically differential topology, with remarkable results [56]. Some of these purely mathematical developments were then in turn used in physics, both QFT and string theories, and M-brain theory. Yang and Mills’s article built on the preceding works on isospin (isotopic spin) in QFT, in which the topology of QFT was cast. Isospin is a “flavor” quantum number, related to the up- and down-quark content of a hadron or meson particle. They develop a nonabelian gauge invariance theory, the main form of QFT leading, via the spontaneous symmetry breaking mechanism, to the Standard Model, containing the electroweak theory and quantum chromodynamics (QCD) that handles the strong force (both based on the quark–gluon model). Both are very complex mathematically but are renormalizable. The Lagrangian of the (minimal) Standard Model is one page long [57] [58] (pp. 166–167). Computers help with calculations, but the complexities of the formalism, even already available or conjectured, let alone those that might be needed in the future, may be beyond reach for us and computers alike. The Lagrangian of QCD, the SU(3) Yang–Mills theory, governing the interaction of quarks and gluons, including confinement, is:

$$L=\sum_{q}({\bar{\psi }}_{qi}i\gamma^{\mu }\left[\delta_{ij}\partial_{\mu }+ig{(G}_{\mu }^{\alpha }t_{\alpha })\right]\psi_{qj}-m_{q}{\bar{\psi }}_{qi}\psi_{qi})-\frac{1}{4}\;G_{\mu \nu }^{\alpha }G_{\alpha }^{\mu \nu }$$

$G_{\alpha }^{\mu \nu }$ is a color field tensor;

$G_{\alpha }^{\mu }$ is a four potential of the gluon field (α=1,…8);

$t_{\alpha }$ are 3×3 Gell-Mann matrices; generators of the SU(3) color group;

$f^{\alpha \beta \gamma }$ are structure constants of the SU(3) color group;

$\psi_{i}$ is the Dirac spinor of the quark field (*i* is a color);

$g=\sqrt{4\pi \alpha_{s\;}}$(ℏ=c=1) is a color charge (strong coupling constant).

The Dirac spinor, ubiquitous in QFT, appears here as well. This Lagrangian might still look elegantly simple to the practitioners of QCD, an elite group. Once, however, one begins calculating, unfolding its symbols, as in David Gross and Frank Wilczek’s or Hugh D. Politzer’s remarkable papers on asymptotic freedom [59,60,61], which brought them their Nobel prize, this elegance retreats, and one enters the calculational nightmares for which QCD and the Standard Model are famous. The dreams of, or yet-undreamed, new theories, perhaps quantum gravity, may yet be born from these nightmares, and the Yang–Mills theory was born from the preceding QFT. It is easy now to forget the immense calculational labor required by the preceding theories of nuclear forces or, for that matter, even QM, as reflected, still very partially, in the above discussion of Heisenberg’s work on QM.

Our hope is the ordering, nearly prohibitively complex as it may be, of the data and formalism. As emphasized above, the essence of quantum physics, both experimental and theoretical, is in its order, rather than randomness, irreducible as the latter may be, beginning with that of quantum correlations, discussed in detail from the RWR perspective [2] (pp. 253–257). Here, I shall focus on the role of symmetry and group theory, especially in QFT. Following the Yang–Mills theory, the local symmetries acquired a special importance as the basis of local gauge theories. A local symmetry preserves a given property when a possibly different symmetry transformation is applicable at different points, thus, in contrast to a global symmetry, making space and space parameters of these transformations. A global symmetry is a local symmetry, but not vice versa. Local symmetries played an important role in the discovery of new particles, such as quarks and gluons, inside the nucleus, and then various types of them, eventually establishing the standard model of particle physics. When QFT predictions concern the effects associated with elementary particles of a given type, such as electrons, photons, and quarks (there are six types), the mathematics of these predictions involves an irreducible representation of the corresponding symmetry group. This is how Murrey Gell-Mann and Georges Zweig (independently) discovered quarks. Each was considering the SU(3) (the noncommutative group of all rotations around the origin in three-dimensional space, represented by three-by-three orthogonal matrices with the determinant equal to one) as the flavor symmetry group for hadrons and noticed that there were no elementary particles associated with the irreducible representations of SU(3). The existence of such particles would imply that hadrons were not elementary particles but composites of new particles, named “quarks” by Gell-Mann and eventually discovered experimentally, along with “gluons,” the bosons mediating the strong force. Neither quarks nor gluons could be observed outside nuclei.

I shall refer to the irreducible representations of symmetry groups corresponding to the elementary particle as “Galois atoms,” following [17]. The concept may echo Heisenberg’s mathematical Platonism insofar as it reflects, first, the role of symmetries in quantum theory and, second, the abandonment of the Democritean atomism of elementary particles. This concept is, however, more in accord with Bohr’s RWR discrete atomism of quantum phenomena, rather than Heisenberg’s materialist Platonism. In RWR interpretations, Galois atoms are only part of the mathematical technology of probabilistic predictions of the data observed in discrete quantum phenomena, rather than, as in Heisenberg’s mathematical Platonism, of purely mathematical representation of the ultimate reality responsible for quantum phenomena. As a mathematical *concept* (although not the term itself), Galois atoms were introduced by Wigner [49], whose work was, as noted, one of the key developments leading Heisenberg to materialist Platonism. I am not saying that “Galois atoms” define (the reality of) elementary particles, even if Heisenberg or Wigner thought so. This claim would be difficult to sustain, in part because we deal with an infinite-dimensional representation of symmetry (Lie) groups in Hilbert spaces, over C, rather than in the actual three-dimensional space, defined over R. This claim is, of course, by definition precluded by RWR interpretations. Heisenberg, too, said that fundamental symmetries *replace* fundamental (elementary) particles, rather than particles, which is a physically defined concept. Wigner’s work was a landmark in the history of symmetries and group representations in quantum theory, in part by virtue of dealing with discrete symmetries, such as mirror symmetries, vs. continuous symmetries, such as those correlative to the standard conservation laws (for momentum, angular momentum, and energy) in accordance with Noether’s theorems. These laws themselves and (suitably modified) Noether’s theorems are, of course, valid in quantum physics [17] (pp. 203–210). The concept of Galois atoms is a great testimony to both the role of abstract mathematics in quantum theory and to the immense reach of Galois’s concept of a group, far beyond the initial problem in algebra that this concept was developed to address. Galois theory is a paradigmatic early concept of abstract algebra and has played a major role in numerous developments of modern mathematics, generally, unlike group theory, removed from physics.

Unexpectedly, Galois theory entered QFT in the context of renormalization, in considering the renormalization group, in A. Connes and M. Marcolli’s and related work [58,62]. This subject will be put aside in view of its formidable technical difficulties. A preliminary conceptual analysis of this approach is offered, under the heading of the Galois principle, by the present author in [63]. I shall merely state, in agreement with Connes and Marcolli, that the role of Galois theory in renormalization suggests that “the divergences [requiring renormalization] of Quantum Field Theory, far from just being an unwanted nuisance, are a clear sign of the presence of totally unexpected symmetries of geometric origin,” symmetries closely tied to the Yang–Mills theory [64] (p. 4075). This fact is yet another testimony to the role of the mathematics of symmetry in QFT and its potential extensions, a role championed by Heisenberg. Already justified by the preceding history of quantum theory, his assessment, in the 1950s, has been amply confirmed by the subsequent development of QFT, from the local gauge symmetries of the Yang–Mills theory to the use of Galois theory in renormalization, and in some new aspects of QM, including quantum information theory (e.g., [65]). This role is likely to remain significant in the future.

## 6. Beyond Atomism and Platonism: The Discrete, the Continuous, and the Unthinkable

This section reconsiders the relationships between continuity and discontinuity in quantum physics, from the standpoint of the RWR view. These relationships, I argue, are likely to play an important role in the future development of fundamental physics, including in the thorny problem of quantum gravity, often seen as the greatest unsolved problem of fundamental physics. The mathematical divergencies of QFT, as manifesting “fundamental symmetries of geometric origins,” rather than a deficiency of QFT, may also indicate the possibility that the use of continuous mathematics in QFT might not be a problem, reflected in the necessity of renormalization of QFT. One of the reasons (which merit a special notice) for these divergencies is the structural reliance, *beyond observation* (as in QM), on the concepts of space and time, or mathematically spacetime, as continuous manifolds in the high-energy QFT. For some, this and related problems have signaled the necessity of an alternative theory based on discrete mathematics. This view, especially in its realist versions, also assumes that the ultimate reality underlying space and time of the current QFT would be discrete at the higher-energy scales, ultimately the Planck scale, potentially making it no longer possible to speak of space or time. The discreteness of this reality is not the same as the discreteness of a theory of this reality, because such a theory may not represent this discrete reality but only predict, possibly probabilistically, as QM or QFT does, effects of this reality, and hence be continuous as a theory. In fact, the discrete ultimate reality underlying space or time has been assumed even in QFT, as it stands now, or even in QM.

The idea of discrete physical theories has been around for a long time [66]. That of the ultimately discrete nature of “the reality underlying space,” or by implication time, in Bernhard Riemann’s phrase (physical space was assumed to be continuous at the time), dating to 1854 [67] (p. 33), has an even longer history, preceding Riemann, and was possibly known to, although not assumed by, Kant. Riemann also noted, on the same occasion, that physics will be different depending on whether this reality is discrete or continuous, and that in the latter case, this geometry, for example, whether it is Euclidean or not, will depend on physics. This was a prophetic insight confirmed by Einstein in GR. Riemann’s ideas were equally influential in the development, soon thereafter, of discrete and finite axiomatic geometries, which came to play an important role in quantum information theory. The idea of spatial discreteness was advocated by Heisenberg from the 1930s on, in part in view of the divergences of QED (with which his S-matrix theory was connected), but only in part and arguably not primarily [1] (pp. 143–145). He remained attracted to this idea after the renormalization of QED and possibly of the QFT (as the Yang–Mills theory) of nuclear forces in 1972, although it is not clear if he still subscribed to the idea by then (he died in 1976). Heisenberg, however, thought of QFT, or his proposed “*World-formula*,” as continuous theories. This continuity was in conflict with his materialist Platonist view insofar as the discrete ultimate reality considered would be represented by continuous mathematics (the problem RWR interpretations avoid). Heisenberg, who must have realized this difficulty, did not address it, perhaps because his proposal concerning the minimal unit of length was hypothetical and, as he said, belonged to future physics, possibly even beyond the *World-formula* [1] (p. 145). Such a future theory could be discrete, thus preserving materialist Platonism by making it discrete as well. This possibility is in accord with subsequently developed and some current proposals, among them, in part as alternatives to string and M-Brane theories (which are continuous), are the loop quantum gravity and causal network theory. These proposals are not motivated, at least not primarily, by the problems of QFT, and will be put aside. The difficulties of QFT form a major motivation for developing discrete alternatives to it, often in conjunction with the discrete nature of the ultimate reality considered. Richard Feynman, who shared (with Julian Schwinger and Sin-Itiro Tomonaga) a Nobel prize for renormalization of QED, reflected on these difficulties, including their arguably bottom line:

It always bothers me that, according to the laws [of quantum physics] as we understand them today, it takes a computing machine an infinite number of logical operations to figure out what goes on in no matter how tiny a region of space, and no matter how tiny a region of time. How can all that be going on in that tiny space? Why should it take an infinite amount of logic to figure out what one tiny piece of space/time is going to do?

[68] (p. 57).

If space and time were discrete, this problem would disappear. There is, however, no discrete mathematics at present to adequately handle quantum phenomena, while QFT is able to do so, its difficulties notwithstanding. As Feynman noted on the same occasion, the problem would also disappear if physics were no longer mathematical, which, as he probably realized, was unlikely to happen and has not in the sixty years since this statement [68] (pp. 57–58). If anything, the intervening decades that witnessed massive mathematical developments in QFT, albeit within the same basic framework, make it even less likely now, while the problem that bothered Feynman is still in place. Developing a discrete mathematics that could replace QFT in its present continuous form is not an easy task, and most attempts in this direction thus far tend to be based on transferring the methods of continuous mathematics to the discrete domain. This is not a criticism. Geometric group theory and Grothendieck étale-cohomology theory, grounded in the concepts of “scheme” and “topos,” in algebraic geometry, which continue to be massively developed, show the effectiveness of this strategy in mathematics, in the second case, even reaching a greater complexity than corresponding continuous theories. It was seen as such by Grothendieck, who compared his contribution to the idea of spatiality, defined by these concepts, to that of Einstein in GR and that of Schrödinger in QM [69] (p. 68). An invocation of Schrödinger is not self-evident. It may, however, be plausibly seen as referring to the role of Hilbert spaces over C in QM, in Schrödinger’s wave mechanics, infinite-dimensional ones, through which one relates to events in actual physical space represented as a three-dimensional real manifold. Schrödinger himself, of course, disparaged QM as “the doctrine born of distress” in his 1935 article, “The present situation in quantum mechanics,” famous for the concept of entanglement and the cat paradox it contains [70] (p. 154). It was Grothendieck’s theory of étale cohomologies, based on Galois theory, that was also one of the motivations for his motive theory, used in the approach to the renormalization group mentioned above [58,62]. Still, while they have earlier precursors in algebra or geometry, such as finite geometries developed in the nineteenth century, these are relatively recent developments. They are competing with centuries or even millennia of the dominance of continuous thinking and mathematics based on it, which led to an immense multitude of powerful theories. In any event, geometrical group theory and étale-cohomology theories are examples, in mathematics, of discrete theories that have a structural complexity comparable to that of the continuous theories dealing with analogous phenomena, such as topological groups or continuous algebraic varieties. One might expect the same for discrete theories in physics in dealing with the phenomena handled by QFT, or still more complex ones, if gravity comes into play.

As Grothendieck noted in connection with Riemann’s view that “the reality underlying space” may be discrete: “it is possible [in reversal of the conventional view] that for the human mind, ‘the continuous’ was easier to grasp than ‘the discontinuous,’ and that it serves us, therefore, as an ‘approximation’ to apprehend the discontinuous” [69] (pp. 67–68). Grothendieck does not explain how this would work in mathematics. On the other hand, QFT allows one to give a sense to this point in physics. The continuous QFTs at their energy scales may only be approximations of the ultimate discontinuous theory at some higher-energy scale.

An innovative proposal of this type, along quantum-informational lines, was advanced in [71,72]. The authors derive both Maxwell’s theory and free Dirac’s equation (in the absence of interaction) as continuous limits of a discrete quantum cellular-automata architecture, defined by a set of fundamental principles, which do not rely on continuity, making the spacetime continuum an emergent phenomenon as well. The approach has an additional mathematical interest in the present context because of its use of geometric group theory. The theory, as noted, emerged from the realization that discrete groups can be considered geometric-like objects and studied by geometric and topological techniques developed for continuous mathematical structures.

RWR interpretations of QFTs comprising the Standard Model avoid the problems of assuming either a continuous or (such assumptions have their problems, too) discrete nature of the ultimate reality responsible for quantum phenomena. The reason is, of course, that in these interpretations, this reality, and as part of the reality underlying the phenomenally observed space and time, is unrepresentable and in strong RWR interpretations, inconceivable. Hence, it cannot be assumed to be discrete any more than continuous, or either spatial or temporal, or in the present (strong RWR) interpretation, more radically, even either material or mental. As I have argued here, however, this does not mean that such a reality does exist either. The concept of this reality is used here as an interpretive assumption in dealing with quantum phenomena by means of quantum theory and not a metaphysical assumption about nature. It is, nevertheless, possible that such a reality does actually exist in the world, or what we so experience, a point discussed below. Accordingly, once an RWR interpretation of quantum phenomena themselves (strictly discrete relative to each other, while precluding any continuous physical connections between them) is in place, it does not matter whether a theory predicting what is observed in these phenomena is continuous or discrete mathematically, insofar as RWR interpretations of this theory are possible. Continuity is only a mathematical feature of the formalism of QM and QFT in their currently standard forms, just as it is that of the formalism of classical physics or relativity. Unlike in the latter theories, however, the continuous mathematics of QM and QFT relates to the data observed as discrete phenomena by predicting the probabilities or statistics of the occurrence of these data. At the same time, each quantum phenomenon is, by the Bohr postulate, represented by classical physics in the continuous space and time, just as observed phenomena are in classical physics or relativity, which are mathematically continuous theories themselves.

That the probabilistic relations between QM or QFT and quantum phenomena are established via the algebra of Hilbert-space operators (cum Born’s rule) gave Heisenberg’s approach an algebraic aspect. Most fundamentally, again, the continuous functions (over R) used in classical physics or relativity (for variables such as position or momentum) are replaced by operators and their algebra in Hilbert spaces over C. On the other hand, qualifying Einstein’s view of “the Heisenberg method” as “purely algebraic” [14] (p. 378), these Hilbert spaces themselves are continuous mathematical objects. They are defined analogously to mathematical spaces that represent physical spaces in classical physics and relativity, with the concept of distance (“norm” in Hilbert spaces) defined algebraically in both cases. As noted, C*-algebras, too, are continuous Banach spaces, and as noncommutative spaces, they are treated by a form of noncommutative topology of measure theory. QFT often deals with Hilbert spaces whose continuity is denser than that of regular continua, such as the (real number) spacetime continuum of classical physics or relativity. Besides, continuous functions are retained, because these Hilbert spaces are those of continuous functions, which are infinite-dimensional vectors when one is dealing with continuous variables, such as “position” and “momentum,” represented by operators. More accurately, these are abstract mathematical elements, *P* and *Q*, that allow one to predict the value, such as the position or the momentum (but never both together because of the uncertainty relations), observed in measuring instruments. In sum, rather than only algebraic, the Heisenberg method was also a new geometrical method, a precursor of noncommutative geometry, in theoretical physics, bringing algebra and geometry together in dealing with the physical discreteness of quantum phenomena. As discussed here, this discreteness is not the same as the Democritean atomism of the limited divisibility of matter. The only “atoms” of QM or QFT in RWR interpretations are Galois atoms manifesting symmetries of these theories. They have no physical but only mathematical reality. In contrast to Heisenberg’s materialist Platonism, however, they have no representational role, but only part of the probabilistically predictive mathematical technology of quantum theory.

Nevertheless, the possibility of discrete quantum theories is part of the present and likely the future of quantum theory. The importance of Heisenberg’s thinking in this regard is not so much in his ideas concerning the discrete nature of the reality underlying space. It is in his way of thinking, defined by the invention of new mathematics from physics. I also suspect that, whether continuous or discrete, any future quantum theory is likely to retain, as its key mathematical feature, the noncommutative structure of quantum theory, one of Heisenberg’s greatest discoveries. As his derivation of QM suggested and as all subsequent quantum theories, from QED to the Yang–Mills theory and the Standard Model, confirmed, this noncommutativity appears to be mathematically unavoidable given the physical structure of quantum phenomena. This mathematics, in effect, that of noncommutative geometry, over C, and its essential relation to probability, via Born’s rule, also bears on the question of quantum gravity, because GR, defined, just as is classical physics, over R, is a commutative and deterministic theory. It is also possible that these two continuous theories are merely two different, incompatible approximations of an as yet unimagined or even unimaginable underlying discrete theory.

## 7. Conclusions

The RWR view of quantum theory may not have ever been that of Heisenberg, even at the time of his invention of QM. Bohr was primarily responsible for the rise of this view, ultimately in its strong form, even if without ever assuming, as this article does, that this reality may even be neither material nor mental, rather than a form of matter. Bohr also saw QM and QFT, including in RWR interpretations, as manifestations of new “harmonies” in our interactions with nature by means of experimental technology and thought, including mathematical thought [3] (v. 3, p. 6). The RWR view was, however, made possible by Heisenberg’s mathematical thinking, leading him to the discovery of QM and its new harmonies, except for entanglement and correlations. The latter were discovered by Schrödinger, with the help of the EPR experiment. Schrödinger, of course, saw these “harmonies” (he would not have seen them as harmonies) as standing in the way of the realist and classical causal harmonies that he, as Einstein, thought should define fundamental physics. In the RWR view, however, the harmonies of quantum physics are the harmonies of probabilities and correlations in our interactions with physical reality, which, as the ultimate reality responsible for quantum phenomena, is beyond the reach of thought. As such, this reality is also beyond any harmony that thought can conceive of. All harmonies, and the very idea of harmony, belong to our thought and are created by it, as were the mathematical harmonies of QM created by Heisenberg’s thought.

As stressed from the outset, the present (RWR) interpretation is an interpretation of quantum phenomena and QM or QFT and does not claim that this kind of reality exists in nature or, because nature is our concept, too, that which is beyond human existence. On the other hand, if, as I argue here, following Lebesgue, who gave us new harmonies of measure theory in mathematics, it is true that that which we cannot conceive of might nevertheless exist, this type of reality *may* exist beyond our own existence. Lebesgue’s insight was prompted by his thinking about the ultimate reality of mathematics, a mental reality. As I have also argued, however, this insight suggests that what we assume (on the basis of our experience and thinking) to be the reality underlying what we experience, commonly seen as mental in mathematics and material in physics, may be neither material nor mental. This may be what quantum physics ultimately tells us about the nature of physical reality. This reality may not be physical or material, but it may not be mental either.

Admittedly, it is impossible to be certain about any of this either. While I close with a stronger *assumption* concerning a possible, *only possible*, nature of reality itself, this article only argues that RWR interpretations of QM or QFT, including the one this article adopts, are logically consistent and are in accord with experimental evidence, as things stand now. This argument leaves space for other, possibly realist, interpretations. Still, even in making this more limited argument, one can only be reasonably confident about it, but not certain, even as things stand now, and manifestly as concerns the future. Only uncertainty is certain when it comes to the ultimate standing and fate of our arguments, just as it is the case, *as things stand now*, in physics, when dealing with quantum phenomena by means of QM and QFT. They do not give and do not promise us certainty in our predictions as classical physics and relativity do, but then QM and QFT only tell us, in physics, about what is possible in the future and not anything about the past, defined by the archived classical world of past measurements. Only the latest of these measurements, in the experimental setup considered, can be used for making new predictions, which are irreducibly probabilistic and hence uncertain, concerning future quantum experiments.

I am not saying that everything is uncertain. As Blaise Pascal said: “It is not certain that everything is uncertain” [73] (Pensee 387). The uncertainty of the future may, however, be our best hope given the precarious state of fundamental physics now, just as, defined by the precarious state of the old quantum theory, it was for quantum physics in 1925, a hope fulfilled, in an unexpected way (and not to everyone’s liking), by Heisenberg’s invention of quantum mechanics. What may be more certain is that, if it comes, this fulfillment will be brought about by the creative force of mathematical thinking, just as the creative force of Heisenberg’s mathematical thinking brought about quantum mechanics.

## Data Availability

No new data were created or analyzed in this study.
